# Descriptions of five new species in *Entoloma* subgenus *Claudopus* from China, with molecular phylogeny of *Entoloma* s.l.

**DOI:** 10.3897/mycokeys.61.46446

**Published:** 2019-12-05

**Authors:** Xiao-Lan He, Egon Horak, Di Wang, Tai-Hui Li, Wei-Hong Peng, Bing-Cheng Gan

**Affiliations:** 1 Soil and Fertilizer Institute, Sichuan Academy of Agricultural Sciences, Chengdu 610066, China Sichuan Academy of Agricultural Sciences Chengdu China; 2 Schlossfeld 17, A-6020 Innsbruck, Austria N/A Innsbruck Austria; 3 State Key Laboratory of Applied Microbiology Southern China, Guangdong Provincial Key Laboratory of Microbial Culture Collection and Application, Guangdong Institute of Microbiology, Guangdong Academy of Sciences, Guangzhou 510070, China Guangdong Institute of Microbiology Guangzhou China

**Keywords:** Entolomataceae, systematics, taxonomy, multi-gene analyses, ecology

## Abstract

Entoloma subgenus Claudopus is widely distributed, yet the taxonomy and systematics of its species are still poorly documented. In the present study, more than forty collections of *Claudopus* were gathered in China and subsequently analysed, based on morphological and molecular data. The results revealed first a high level of species diversity of *Claudopus* in China and second, there is a wide ecological range regarding the substrates and the habitats ranging from temperate, tropical to subalpine locations. Based on morphological and molecular evidence, five novel species from China are proposed, viz. *E. conchatum*, *E. flabellatum*, *E. gregarium*, *E. pleurotoides* and *E. reductum*. Molecular phylogeny of *Entoloma* s.l. was also reconstructed, based on 187 representatives of *Entoloma* s.l. by employing the combined ITS, LSU, mtSSU and RPB2 sequences. Ten monophyletic clades (*Claudopus*, *Leptonia*, *Nolanea*, Cuboid-spored *Inocephalus*, “*Alboleptonia*”, *Cyanula*, *Pouzarella*, *Rhodopolia*, *Prunuloides* and *Rusticoides*) were recovered, while 13 taxa could not be placed in any defined clades. The results confirmed that *Claudopus* in a traditional morphological sense is not monophyletic and the *Rusticoides*-group, previously considered within *Claudopus*, formed a separate clade; but section Claudopus and relatives of *E. undatum* belong to a distinctive monophyletic group. Despite some monophyletic groups in *Entoloma* s.l. being distinctive in both morphology and molecular phylogeny, they were still treated as subgenera of *Entoloma* s.l. temporarily, because accepting them as genera will make *Entoloma* s.l. paraphyletic.

## Introduction

*Entoloma* P. Kumm. is a large agaric genus and more than 1000 species have been reported worldwide. Subgenus Claudopus is one of the most distinctive groups in this genus. By comparison, the pleurotoid or omphalinoid basidiomes of all described species of *Claudopus* are small and, accordingly, are often overlooked and thus neglected during field work. Macromorphologically, members of *Claudopus* are characterised by the fibrillose, non-gelatinised pileipellis and the eccentric, lateral or absent stipe. Macroscopically, fresh basidiomes of *Claudopus* are readily confused with species belonging to genera *Clitopilus* (Fr. ex Rabenh.) P. Kumm., *Crepidotus* (Fr.) Staude, *Hohenbuehelia* Schulzer, *Marasmiellus* Murrill or *Pleurotellus* Fayod but, under the microscope, the identification of *Claudopus* is immediately confirmed by the typical angular basidiospores.

To date, the taxonomic position of *Claudopus* (and of other entolomatoid taxa) is still controversial and unresolved. Horak (1980, 2008) accommodated pleurotoid *Claudopus* at generic level. Largent (1994) proposed 13 entolomatoid genera, including *Claudopus* as a recognised genus. Noordeloos (1992, 2004) proposed that *Claudopus* should be included in the genus *Entoloma*. All these proposals were based on morphological characters. However, molecular analysis has substantially altered traditional concepts in many fungal groups, showing that the taxonomic delimitation, based on morphological characters alone, is in fact artificial and speculative (Li et al. 2011; Chen et al. 2016; Han et al. 2016). In recent years, molecular markers have also been employed in phylogenetic studies on entolomatoid groups. In a paper by Co-David et al. (2009), three *Claudopus* species are nested in the monophyletic *Nolanea*-*Claudopus* Clade. As a consequence, the authors concluded that the traditional concept of *Claudopus* is polyphyletic. However, it was obvious that the number of samples was too limited to support the placement of *Claudopus* within *Nolanea*. Two subsequent molecular phylogenetic studies have also demonstrated that *Claudopus* (only pleurotoid species and relatives of *E. undatum* (Gillet) M.M. Moser were included) was grouped with *Nolanea* and *Leptonia* members, but no significant support was received (Baroni and Matheny 2011; Kinoshita et al. 2012). However, it is noteworthy that, in these studies, *Claudopus* species were actually nested in a monophyletic subclade (Baroni and Matheny 2011; Kinoshita et al. 2012). In two more recent studies (Vila et al. 2014; He et al. 2015), the results suggested that section Claudopus and *E. undatum* complex species could belong to a monophyletic group. Additionally, *E. rusticoides* (Gillet) Noordel. and associated taxa in sect. Undatum (Noordeloos 2004) are rather distant from the monophyletic *Claudopus* lineage (Vila et al. 2014). However, in all the previous studies, limited taxa of *Claudopus* were included to support these conclusions and we expect that the topologies of the phylogenetic trees will probably significantly change with the increase in samples taken into account.

Based on Index of Fungi, the reference for about 70 species of “*Claudopus*” (both at subgeneric or generic level, including the *rusticoides*-group) were found in the pertinent literature. Substantial contributions were published, for example, by Dennis (1970), Esteve-Raventos and De La Cruz (1998), Horak (1980, 2008), Largent (1976, 1994), Largent et al. (2011), Manimohan et al. (2002, 2006), Noordeloos (1981, 1992, 2004), and Pegler (1983). Recently, two Chinese *Claudopus* species were added to the list (Deng et al. 2015; He et al. 2015). However, *Claudopus* worldwide still remains poorly understood and, for many species, no additional records have been added since the first publication.

There are several reasons why the taxonomy of *Claudopus* worldwide is not well understood yet. For many species, the original descriptive documentation is inadequate and/or type material is either not extant or in poor condition (Horak, unpubl. data). In addition, the knowledge of macroscopic and microscopic characters of nearly all described species is limited, at least by comparison with taxa of *Entoloma* s.l. The colour of the young basidiomes ranges from white, grey to pale brown. Bluish colours are the exception and are observed only in a few species (Horak 1980; Manimohan et al. 2002; Morozova et al. 2012). As a rule, the majority of the original descriptions are relying on macromorphological features which have led to persisting confusion regarding the circumscription of taxa. Furthermore, the number of reliable microscopical characters (basidiospores, pileipellis structure) is restricted. Distinctive cheilocystidia are not found in most species of *Claudopus*. Presence or absence of clamp connections (in particular at the basal septum of basidia) have not been given adequate attention in many of the earlier descriptions. Only recently, few species of *Claudopus* have been described by combining both morphologic and molecular data (Largent et al. 2011; Vila et al. 2014; Deng et al. 2015; He et al. 2015). Accordingly, identification of *Claudopus*, based on both morphological characters and molecular markers, will be helpful to fill a gap in our knowledge of *Claudopus* and bring clarifications regarding taxonomic relationships in this group.

To better understand the phylogeny of the genus *Entoloma* s.l. and the placement of *Claudopus* in this genus, a more comprehensive molecular phylogeny of *Entoloma* s.l. was carried out by employing the combined ITS, LSU, mtSSU and RPB2 sequences in the present paper. On the other hand, both descriptive and molecular information about 5 new taxa in subgenus Claudopus recently discovered at various localities in China is provided and the first record of Entoloma byssisedum var. microsporum Esteve-Rav. & Noordel. (originally reported from Spain) is listed for the Chinese mycota. Key and descriptions also take the recently described Chinese *Claudopus* species into consideration, viz. *E. crepidotoides* W.Q. Deng & T.H. Li and *E. alpinum* Xiao L. He, W.H. Peng & B.C. Gan (Deng et al. 2015; He et al. 2015). It can be assumed that the number of Chinese representatives of *Claudopus* will significantly increase with further fieldwork.

## Material and methods

### Morphological descriptions

Photographs of fresh specimens are taken *in situ* and all relevant ecological data are recorded at the actual habitat. All macromorphologic descriptions are also based on fresh material. Colour notations follow Kornerup and Wanscher (1978). Micromorphologic data are sketched with the help of a drawing tube attached to a Wild M 20 microscope. Basidiospores, basidia and pileipellis were mounted and measured in 5% potassium hydroxide (KOH) and/or 1% Congo Red. Measurements of the basidiospores exclude hilar appendix (apiculus). Q is used to mean “length/width ratio” of a basidiospore in profile view; ± means sample standard deviation; ***Q*** means average Q of all basidiospores; *x* means of basidiospore length and width. All holotype collections are kept in the Mycological Herbarium of Soil and Fertilizer Institute, Sichuan Academy of Agricultural Sciences (**SAAS**); isotypes and duplicates are preserved in Herbarium ZT, ETH, Zurich, Switzerland.

### DNA extraction, PCR amplification and sequencing

Procedures of Genomic DNA extraction, PCR amplification, PCR products purification and sequencing were the same as in previous studies (He et al. 2013). The primers for RPB2 amplification were rpb2-6f and rpb2-7r, rpb2-i6f and rpb2-i7r (Co-David et al. 2009). The ITS regions were amplified with primer pairs ITS5 and ITS4 (White et al. 1990). The nLSU regions were amplified with primer pairs LR0R and LR5 (http://www.biology.duke.edu/fungi/mycolab/primers.htm). The mtSSU regions were amplified with primer pairs MS1 and MS2 (White et al. 1990).

### Sequence alignment and phylogenetic analyses

Phylogenetic analyses were carried out, based on the ITS dataset and the combined dataset of ITS, nLSU, RPB2 and mtSSU. Sequences used in analysis are listed in table 1 and aligned in muscle 3.6 (Edgar 2004). If necessary, the aligned sequences were manually modified employing BioEdit 7.0.9.0 (Hall 1999). Representative sequences, published in previous studies, focused on entolomatoid fungi and new sequences, generated in this study, were selected for the present molecular analyses (Co-David et al. 2009; Baroni et al. 2011; Baroni and Matheny 2011; Largent et al. 2011; Kinoshita et al. 2012; Morgado et al. 2013; Morozova et al. 2014; Vila et al. 2014; He et al. 2015; Kokkonen 2015). The quality of these sequences was further accessed and those sequences in low quality were excluded in the present study. Almost all representative species in these studies were included. No conflicts between the ITS, nLSU, RPB2 and mtSSU datasets were observed by comparing the topologies resulting from the phylogenetic analysis of the single gene and therefore they were combined in the analysis.

Maximum Likehood (ML), Maximum Parsimony (MP) and Bayesian analyses were performed on the combined dataset, respectively. ML analyses were carried out by the web RAxML Version 8 (http://www.phylo.org/sub_sections/portal/) under the GTR+G+T model with 1000 bootstrap replicates (Stamatakis 2014). “Find best tree using maximum likelihood search” option was selected when analysing. MP analyses was performed using PAUP* version 4.0b10 (Swofford 2003). All characters were treated as unordered and of equal weight. Gaps were treated as missing data. Bootstrap values (BS) were calculated from 1000 replicates. Bayesian analyses were performed using MrBayes 3.2.6 (Ronquist and Huelsenbeck 2003). The best substitution models for each marker were selected by using Akaike Information Criterion (AIC) in jModelTest 2.1.7 (Darriba et al. 2012). GTR+I+G model was employed for nLSU, mtSSU and ITS, SYM+G for RPB2. Six Markov chains were run for 2 runs from random starting trees for 15 million generations and sampled every 100 generations. Average standard deviation of split frequencies below 0.05 is an indication of convergence. Bayesian Posterior Probabilities (BPP) were determined after calculating a 75% majority rule consensus tree.

## Results

### Taxonomy

#### 1. Entoloma conchatum

Taxon classificationFungiAgaricalesEntolomataceae

Xiao L. He & E. Horak sp. nov.

E6291891-4395-5150-A871-63B321DDCF0B

817515

Figures 1a, b , 2

##### Type.

China. Sichuan Prov., Miyi County, Huangqiao reservoirs, ca. 1500 m elev., 26°42'–27°10'N, 101°41'–102°15'E, on soil, 13 September 2015, X.L. He (*SAAS 1712*, holotype; *ZT 13628*, isotype).

##### Sequences ex holotype.

KU312111 (ITS), KU534220 (nLSU), KU534459 (RPB2), KU534432 (mtSSU).

##### Etymology.

*conchatum* (Lat.), referring to the conchate shape of the basidiomes.

##### Diagnosis.

*Entoloma conchatum* closely resembles *Entoloma parasiticum* (Quél.) Kreisel, described from Europe and N-America but differs by smaller basidiospores. The Australian *C. viscosus* is separated from *E. conchatum* by its sticky pileus and the absence of rhizoids at the base of the rudimentary stipe.

Pileus 7–15 mm, conchate, broadly convex, at first white, becoming orange-white, yellowish-white and finally pale pinkish in age, entirely matted-tomentose to matted-depressed fibrillose, opaque, dry, not hygrophanous, margin not transparent-striate. Lamellae with 2–4 tiers of lamellulae, adnexed, up to 2 mm wide, subventricose, subdistant, white at first, becoming pinkish in age, entire margin concolorous. Stipe 1–3 × 0.5–1 mm, lateral, strongly reduced, covered with minute, white fibrils, base with white mycelium. Rhizoids absent. Context white, thin, unchanging. Odour and taste not distinctive.

Basidiospores 8–10 (10.5) × (6) 6.5–8 μm (*x* = 9.0 ± 0.3 × 7.3 ± 0.3 μm), Q = 1.2–1.4, ***Q*** = 1.28 ± 0.03, 5–6-angled, heterodiametric in profile view. Basidia 28–34 × 9–12 µm, subclavate, 4-spored (also often 2-spored). Lamellar edge fertile. Cheilocystidia, pleurocystidia and caulocystidia absent. Pileipellis a cutis composed of cylindrical hyphae, terminal cells (25–)35–50 × 4–7 µm, cylindrical (or slender subclavate, weakly gelatinised wall thin, smooth or minutely encrusted with pale yellow pigment. Oleiferous hyphae numerous in pileipellis. Clamp-connections present in all tissues.

##### Habitat.

Amongst moss on stem base of living conifers and fallen branches of conifers (*Pinus* sp., *Picea* sp.), or on roadside in conifers forest or broadleaf forest.

##### Additional materials examined.

China. Sichuan Prov., Miyi County, Huangqiao Reservoirs, ca. 1500 m elev., 26°42'N, 101°41'E, on soil, 13 September 2015, *X.L. He* (*SAAS 1415*); on fallen branches of conifers, 13 September 2015, *X.L. He* (*SAAS 1117*); X.L. He (*SAAS 1378*); amongst moss on stem base of living conifers, 13 September 2015, *X.L. He* (*SAAS 1470*); on soil, 13 September 2015, *X.L. He* (*SAAS 1014*; *ZT 13609*; *SAAS 1364*; *ZT 13615*). Yunnan Prov., Jinghong County, Dadugang, ca. 1200 m elev., 22°30'N, 101°45'E, on soil, 27 August 2011, *X.L. He and M. Zhang* (*GDGM 28817*).

##### Remarks.

*Entoloma conchatum* is characterised by basidiomes gradually changing colour from white to pinkish, matted-fibrillose pileus, 5–6-angled basidiospores and presence of clamp connections.

Macromorphologically (white fibrillose basidiomes), the following taxa resemble *E. conchatum* viz. *E. crepidotoides* W.Q. Deng & T.H. Li, recently described from tropical China, *E. indocarneum* Manim., Leelav. & Noordel. from India, *E. exiguum* Esteve-Rav. & M. de la Cruz, *E. jahnii* Wölfel & Winterh. and *E. parasiticum* from Europe, *Claudopus minutoincanus* Largent & Abell-Davis, *E. pitereka* Noordel. & G.M. Gates, *C. rupestris* Largent & Abell-Davis and *C. viscosus* Largent & Abell-Davis from Australia and, finally, *C. pandanicola* E. Horak from Papua New Guinea. However, *E. jahnii*, *E. exiguum* and *E. parasiticum* are readily distinguished by the much larger basidiospores (9.4–12 × 6.4–8.3 µm, 9.5–12.5 × 8–10.5 µm, 9.7–12.9 × 7.6–10.2 µm, respectively; Esteve-Raventos & De La Cruz 1998; Noordeloos 1992, 2004). *Entoloma pitereka* differs in the prominent basal rhizomorphs and larger basidiospores (8–12 × 6–8 µm, Noordeloos and Gates 2012); *C. pandanicola* is separated by the translucent striate pileus and smaller basidiospores (7–8 × 6.5–7.5 µm, Horak 1980). *C. minutoincanus* is different from *E. conchatum* in the sticky pileus and absence of clamp connections; *C. rupestris* is separated by the 4–5-angled and smaller basidiospores (6.5–9.2 × 6–8 µm) and absence of clamp connections; *C. viscosus* is distinctive by the presence of rhizoids and absence of clamp connections (Largent et al. 2011). The only white *Claudopus* species recently described from tropical China, *E. crepidotoides*, is recognised by the smaller basidiospores (8–9 × 6–7 µm, Deng et al. 2015). *E. indocarneum* (as *E. carneum* Manim., Leelav. & Noordel. in Manimohan et al. 2002) is readily distinguished from *E. conchatum* by its smooth pileus, presence of mycelial rhizoids and narrower basidiospores (7.5–10 × 5–7 µm, Manimohan et al. 2002).

**Figure 1.**
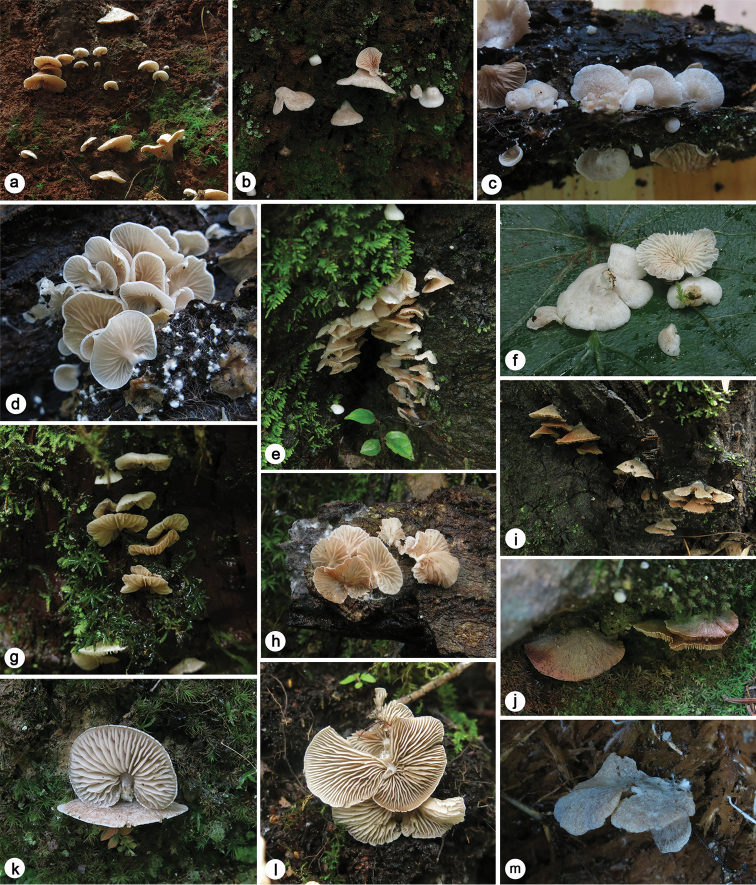
Basidiomes of *Claudopus* species **a** Basidiomes of *E. conchatum* on soil (SAAS 1712) **b** Basidiomes of *E. conchatum* on stem of live *Pinus* (SAAS 1014) **c** Pileus of *E. flabellatum* (SAAS 1501) **d** Lamellae of *E. flabellatum* (SAAS 1080) **e** Basidiomes of *C. gregarious* on bark-wood of live *Castanopsis* (SAAS 1220) **f** Red droplets on the lamellar edges of *E. gregarium* (SAAS 1493) **g** Basidiomes of *E. pleurotoides* on decaying bark-wood of *Castanopsis* (SAAS 1215) **h** Basidiomes of *E. pleurotoides* on bark-wood of live *Castanopsis* (SAAS 1252) **i** Basidiomes of *E. reductum* on decaying stump of *Castanopsis* (holotype, SAAS 1091) **j** Mature basidiomes of *E. reductum* on rock (SAAS 2068) **k** Young basidiomes of *E. reductum* on soil (SAAS 1016) **l** Lamellae of E. byssisedum var. microsporum (SAAS 1828) **m** Basidiomes of E. byssisedum var. microsporum on decaying stump of *Betula* (SAAS 1160).

**Figure 2.**
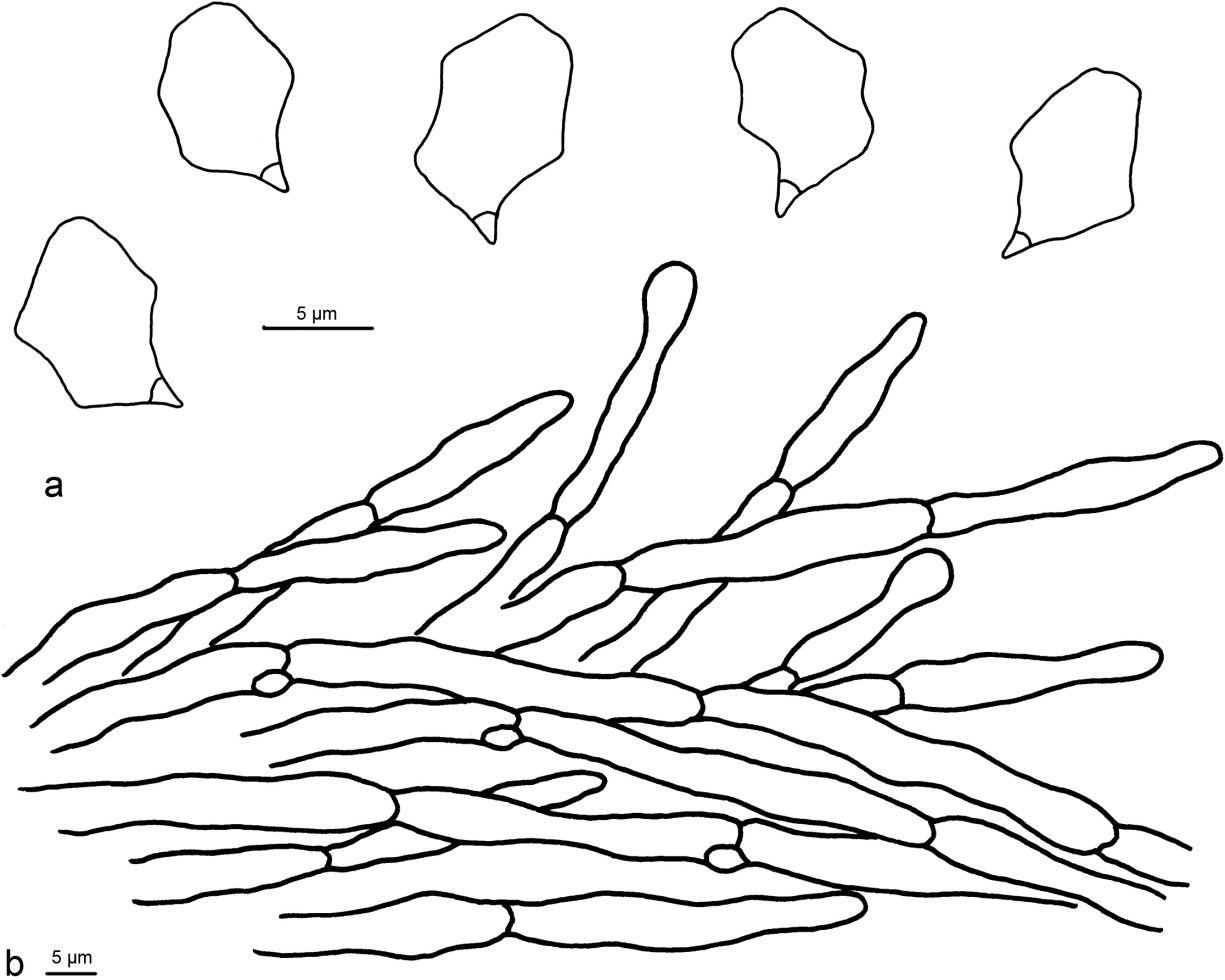
Microscopic structures of *Entoloma conchatum* (holotype) **a** Basidiospores **b** Basidia **c** Pileipellis.

#### 2. Entoloma flabellatum

Taxon classificationFungiAgaricalesEntolomataceae

Xiao L. He & E. Horak sp. nov.

2A16A9B8-E65B-5D10-846D-9B0B0BAAD03D

817516

Figures 1c, d , 3

##### Type.

China. Guizhou Prov., Leishan County, Leigong Mountain, ca. 1600 m elev., 26°22'N, 108°12'E, on decaying stump of fagalean tree, 19 July 2014, *X.L. He* (*SAAS 1080*, holotype; *ZT 13612*, isotype).

##### Sequences ex holotype.

KU312115 (ITS), KU534217 (nLSU), KU534470 (RPB2).

##### Etymology.

*flabellatum* (Lat.), referring to the fan-like shape of the basidiomes.

##### Diagnosis.

*Entoloma flabellatum* is separated from the sympatric *E. pleurotoides* by the more heterodiametric basidiospores.

Pileus 5–15 mm, conchate, broadly convex, becoming applanate with age, entirely matted-tomentose to matted-appressed fibrillose, membranous, whitish at first, becoming orange-white, yellowish-white to pale pinkish with age, weakly hygrophanous, non-striate to minutely sulcate-striate towards margin, dry. Lamellae 6–15, with 2–3 tiers of lamellulae, adnexed, distant, narrow, ventricose, up to 1.5 mm wide, white at first, becoming pinkish with age, entire edges concolorous. Stipe 1–2.5 × 0.5–1 mm, lateral, strongly reduced, pale grey-brown, covered with minute pale grey fibrils, base with white mycelium and prominent white rhizoids. Context thin, unchanging. Odour and taste not distinctive.

Basidiospores 8–10.5 (11) × 6–7 (7.5) µm (*x* = 8.9 ± 0.3 × 6.4 ± 0.3 µm), Q = 1.26–1.52, ***Q*** = 1.38 ± 0.04, 5–7-angled, heterodiametric in profile view. Basidia 28–38 × 7–8 µm, slender clavate, 4-spored, clampless. Lamellar edge fertile. Cheilocystidia, pleurocystidia and caulocystidia absent. Pileipellis a cutis composed of cylindrical hyphae, terminal cells (25–) 30–80 × 6–10 µm, repent or slightly uplifted, subclavate, non-gelatinised walls thin, smooth or minutely encrusted with yellowish pigment, subpellis composed of short-celled cylindrical hyphae, 6–20 µm diam. Oleiferous hyphae absent. Clamp connections present in pileipellis.

##### Habitat.

On decaying stump of fagalean tree, in bamboo forest.

##### Additional materials examined.

China. Guizhou Prov., Leishan County, Leigong Mountain, ca. 1600 m elev., 26°22'N, 108°12'E, on decaying stump of fagalean tree, 18 July 2014, *X.L. He* (*SAAS 1501*, *ZT 13605*).

##### Remarks.

*Entoloma flabellatum* is distinguished by the small and pleurotoid basidiomes, prominent white rhizoids attached to the rudimentary lateral stipe and (5–) 6-angled basidiospores measuring 7.5–8.5 × 5.5–6.5 µm. The basidiomes are white at first but gradually change to yellowish or orange with age.

Numerous species of *Claudopus* recorded from SE-Asia and Australasia are characterised by white basidiomes, cf. Horak (1980), Manimohan et al. (2002), Noordeloos (2004), Largent et al. (2011) and Noordeloos and Gates (2012). The Chinese *E. flabellatum* closely resembles the following three taxa recently described from Australia viz. *E. pitereka* which differs by larger basidiospores (8–12 × 6–8 µm, Noordeloos and Gates 2012), spermatic odour and nutty taste. In addition, the ecology of *E. flabellatum* and *E. pitereka* differs distinctly: *E. pitereka* is reported to occur on rotten wood-bark in wet *Eucalyptus* forest, while *E. flabellatum* is found on rotten wood-bark in subtropical bamboo forest of SW-China. *C. minutoincanus* is also similar to the Chinese *E. flabellatum*, but is distinguished by more isodiameteric basidiospores (7.4–11.4 × 6.3–9.6 μm, Q = 1.08–1.44, Largent et al. 2011). In addition, the RPB2 sequence of *E. flabellatum* is 97% identical to that of *C. minutoincanus*, indicating that they are closely related, but separate species. Macroscopically and microscopically *C. viscosus* is difficult to separate from *E. flabellatum*. However, apart from different size of the basidiospores, the RPB2 sequences of the two species have no significant molecular similarity and thus indicate to represent different and not closely related species (Fig. 9).

**Figure 3.**
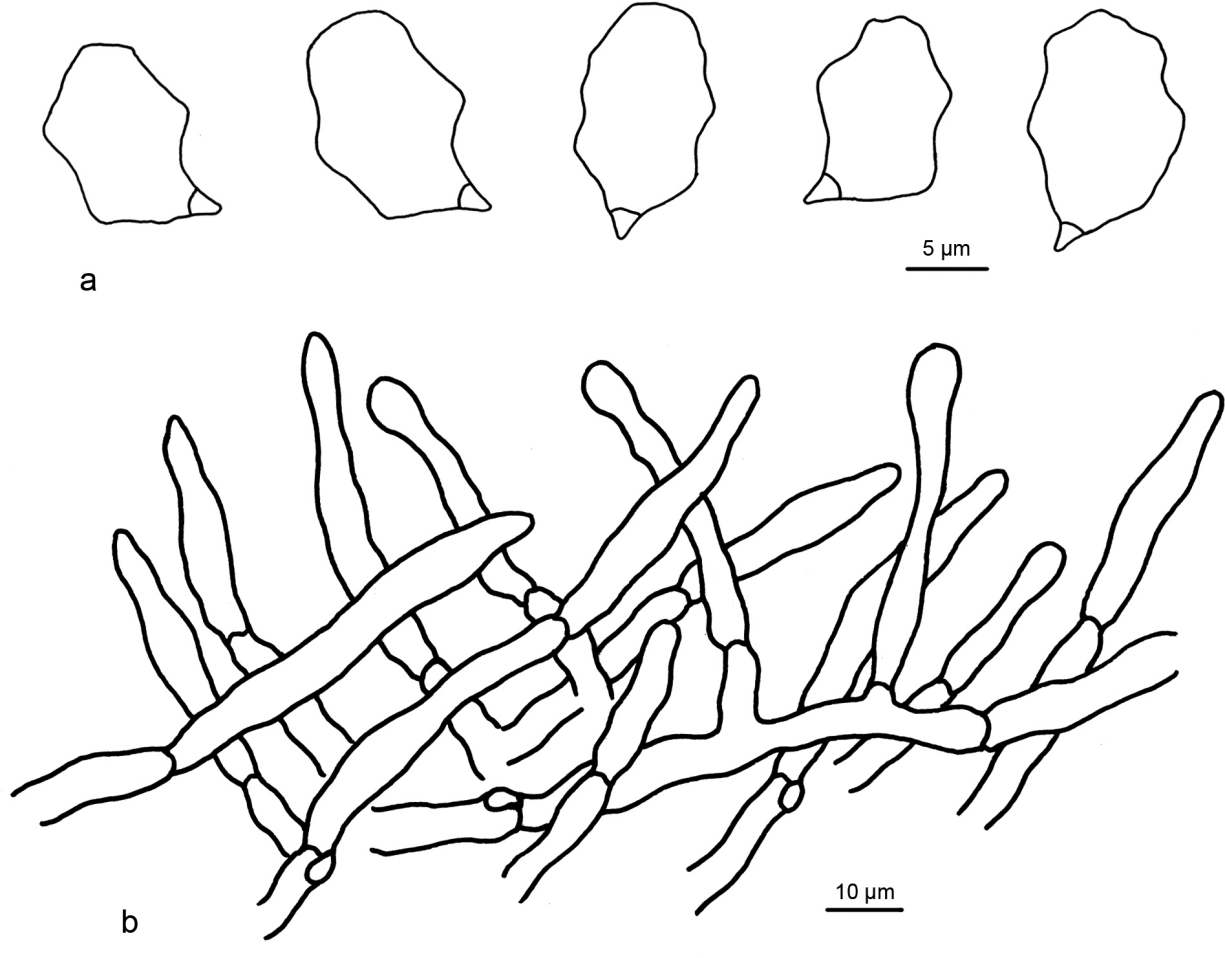
Microscopic structures of *Entoloma flabellatum* (holotype) **a** Basidiospores **b** Basidia **c** Pileipellis.

#### 3. Entoloma gregarium

Taxon classificationFungiAgaricalesEntolomataceae

Xiao L. He & E. Horak sp. nov.

D6E4A9D3-A31F-5CD2-9CB9-F86F0D0DF17B

817517

Figures 1e, f , 4

##### Type.

China. Yunnan Prov.: Binchuan County, Jizu Mountain, ca. 2700 m elev., 25°58'N, 100°21'E, on stem base of living *Castanopsis*, 8 September 2015, *X.L. He* (*SAAS 1220*, holotype).

##### Sequences ex holotype.

KU312122 (ITS), KU534237 (nLSU), KU534474 (RPB2), KU534423 (mtSSU).

##### Etymology.

*gregarium* (Lat.), referring to gregarious habit.

##### Diagnosis.

*Entoloma gregarium* resembles the Chinese *E. conchatum*, but differs by smaller basidiospores.

Pileus 5–10 mm, conchate, broadly convex, pure white, unchanging with age, entirely matted-tomentose to matted-depressed fibrillose, opaque, dry, not hygrophanous, margin not striate. Lamellae adnexed, subdistant to distant, subventricose, up to 2 mm wide, with two tiers of lamellulae, white at first, becoming pale pink, in moist condition with small red droplets at edges. Stipe 1–3 × 0.5–1 mm, strongly reduced, lateral, translucent, covered with minutely, white fibrils, equal, with white basal mycelium. Context white, unchanging, thin. Odour and taste not distinctive.

Basidiospores 7–9 (9.5) × 5.5–7 µm (*x* = 7.7 ± 0.3 × 6.3 ± 0.3 µm), Q = 1.16–1.47, ***Q*** = 1.25 ± 0.04, 5–6 (7)-angled, heterodiametric in profile view. Basidia (26–) 30–34 × 7–10 µm, subclavate, 4-spored, clampless. Lamellar edge fertile. Cheilocystidia, pleurocystidia and caulocystidia absent. Pileipellis a cutis of cylindrical hyphae, terminal cells (25–) 35–60 × 5–10 µm, subclavate or cylindrical (rarely also subfusoid), repent or slightly uplifted, non-gelatinised wall thin, smooth, with inconspicuous plasmatic pigment, subpellis composed of short-celled cylindrical hyphae, 6–14 µm diam. Oleiferous hyphae present in pileipellis. Clamp-connections present in all tissues.

##### Habitat.

Amongst moss on stem base of living *Castanopsis* in fagalean forest.

##### Additional materials examined.

China. Yunnan Prov.: Binchuan County, Jizu Mountain, ca. 2700 m elev., 25°58'N, 100°21'E, on stem base of living *Castanopsis*, 8 September 2015, *X.L. He* (*SAAS 1493*); *X.L. He* (*SAAS 1535*).

##### Remarks.

As compared to other sympatric Chinese species, *E. gregarium* is unique due to the combination of the following characters viz. persistently white and gregarious basidiomes and small basidiospores.

The aforementioned taxa of *Claudopus* viz. *E. conchatum*, *E. indocarneum*, *E. crepidotoides*, *E. exiguum*, *E. jahnii*, *C. minutoincanus*, *C. pandanicola*, *E. parasiticum*, *E. pitereka*, *C. rupestris* and *C. viscosus* have white basidiomes and, accordingly, are macroscopically similar to *E. gregarium*. However, *E. gregarium* is separated from *E. conchatum*, *E. jahnii*, *C. minutoincanus*, *E. parasiticum*, *E. pitereka* and *C. viscosus* by smaller basidiospores; *C. rupestris* differs by the 4–5-angled basidiospores (Largent et al. 2011; Noordeloos 2004).

Based on macromorphological characters, *E. gregarium* is difficult to distinguish from *E. crepidotoides* (Deng et al. 2015); however, the different habitats allow the two species to be discriminated. Additionally, the molecular evidence (Figs 8, 9) of *E. crepidotoides* and *E. gregarium* clearly indicate that they are two distinctive species. *E. indocarneum* is characterised by smooth pileus and presence of mycelial rhizoids (Manimohan et al. 2002). *Claudopus pandanicola*, originally described from tropical Papua New Guinea, is separated by the striate pileus and the different shape of the basidiospores (7–8 × 6.5–7.5 µm, Horak 1980).

**Figure 4.**
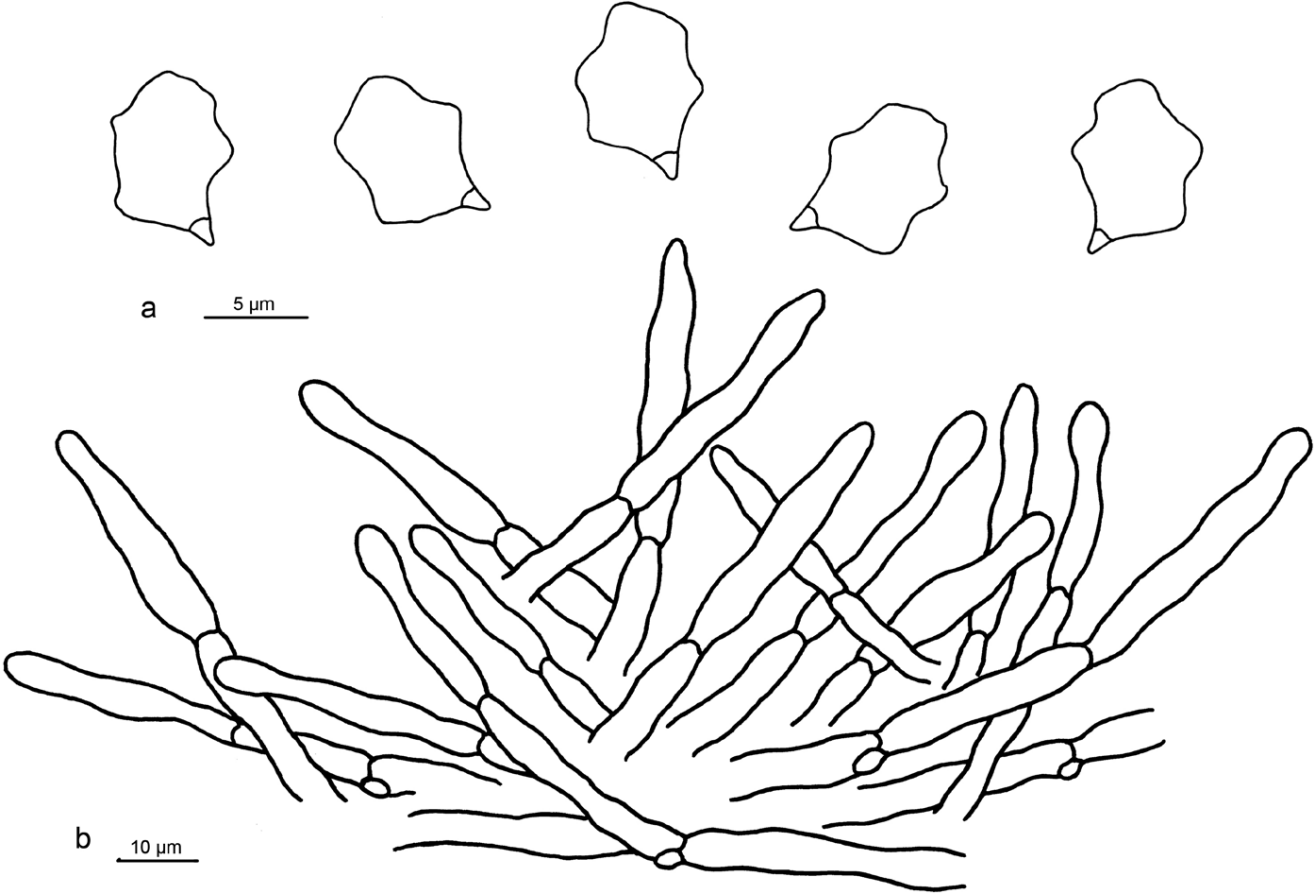
Microscopic structures of *Entoloma gregarium* (holotype) **a** Basidiospores **b** Basidia **c** Pileipellis.

#### 4. Entoloma pleurotoides

Taxon classificationFungiAgaricalesEntolomataceae

Xiao L. He & E. Horak sp. nov.

B9B8F01A-695B-51F0-9DB5-80D1B359E829

817514

Figures 1g, h , 5

##### Type.

China. Yunnan Prov.: Jingdong County, Ailao Mountain, ca. 2500 m elev., 24°23'N, 100°47'E, amongst moss at base of living *Castanopsis* sp., 10 September 2015, *X.L. He* (*SAAS 1252*, holotype; *ZT 13610*, isotype).

##### Sequences ex holotype.

KU312113 (ITS), KU534227 (nLSU), KU534468 (RPB2), KU534424 (mtSSU).

##### Etymology.

*pleurotoides* (Lat.), referring to the pleurotoid shape of the basidiomes.

##### Diagnosis.

*Entoloma pleurotoides* is close to the Australian *E. pitereka*, but differs by smaller and more isodiameteric basidiospores.

Pileus 5–15 mm, conchate, broadly convex, becoming applanate with age, entirely matted-tomentose to matted-appressed fibrillose, membranous, whitish at first, becoming orange-white, yellowish-white and finally pale pinkish with age, slightly hygrophanous, margin not transparent-striate. Lamellae 7–11, with 1–2 tiers of lamellulae, adnexed, distant, narrow, up to 1.5 mm wide, subventricose, white at first, becoming pinkish with age, entire edges concolorous. Stipe 1–2.5 × 0.5–1 mm, strongly reduced, lateral, pale grey brownish, covered with minutely, pale greyish fibrils, base with white mycelium, white basal rhizoids present. Context thin, unchanging. Odour absent. Taste not distinctive.

Basidiospores 8–10 × (7) 7.5–9.5 µm (*x* = 9.2 ± 0.2 × 8.4 ± 0.3 µm), Q = 1.0–1.25, ***Q*** = 1.1 ± 0.03, 5–6-angled, isodiametric to subisodiametric, 5–6-angled in profile view, with pronounced angles. Basidia 32–40 × 12–14 µm, clavate, 4-spored, clampless. Lamellar edge fertile. Cheilocystidia, pleurocystidia and caulocystidia absent. Pileipellis a cutis composed of cylindrical hyphae, terminal cells (25–) 30–40 × 3–8 µm, subclavate or cylindrical (rarely also subfusoid), repent or slightly uplifted, non-gelatinised wall thin, smooth, with inconspicuous plasmatic pigment, subpellis composed of short-celled cylindrical hyphae, 5–10 µm diam. Oleiferous hyphae present in pileipellis. Clamp-connections present.

##### Habitat.

Amongst moss at base of living *Castanopsis* sp. or on decaying debris of *Castanopsis* sp.

##### Additional materials examined.

China. Yunnan Prov.: Jingdong County, Ailao Mountain, ca. 2500 m elev., 24°23'N, 100°47'E, amongst moss at base of living *Castanopsis* sp., 10 September 2015, *X.L. He* (*SAAS 1354*); on decaying debris of *Castanopsis* sp., 10 September 2015, *X.L. He* (*SAAS 1215*; *ZT 13613*); Wuliang Mountain, ca. 2200 m elev., 24°45'N, 100°30'E, amongst moss at base of living *Castanopsis* sp., 9 September 2015, *X.L. He* (*SAAS 1007*).

##### Remarks.

*Entoloma pleurotoides* is characterised by white, small and pleurotoid basidiomes, presence of basal rhizoids and isodiametric to subisodiametric basidiospores.

Macromorphologically, *E. pleurotoides* closely resembles *E. pitereka* which, however, differs by more heterodiametric basidiospores (Noordeloos and Gates 2012). Two other Australian species of *Claudopus* (*C. rupestris*, *C. viscosus*) possess basal rhizoids but, contrary to *E. pleurotoides*, are recognised by 4–5-angled, heterodiametric basidiospores (Largent et al. 2011). Basal rhizoids are also reported for the Indian *E. indocarneum* (Manimohan et al. 2006), which is separated by smooth pileus and more pronounced heterodiametric basidiospores (7.5–10 × 5–7 µm, Manimohan et al. 2002). Concerning macromorphologic characters, *E. pleurotoides* is difficult to separate from the following *E. flabellatum* discovered in China; however, the latter differs by the more distinctive heterodiametric basidiospores. In addition, molecular evidence further confirms that the two taxa represent two defined species. The ITS sequences of *E. flabellatum* are 88% identical to those of *E. pleurotoides*.

**Figure 5.**
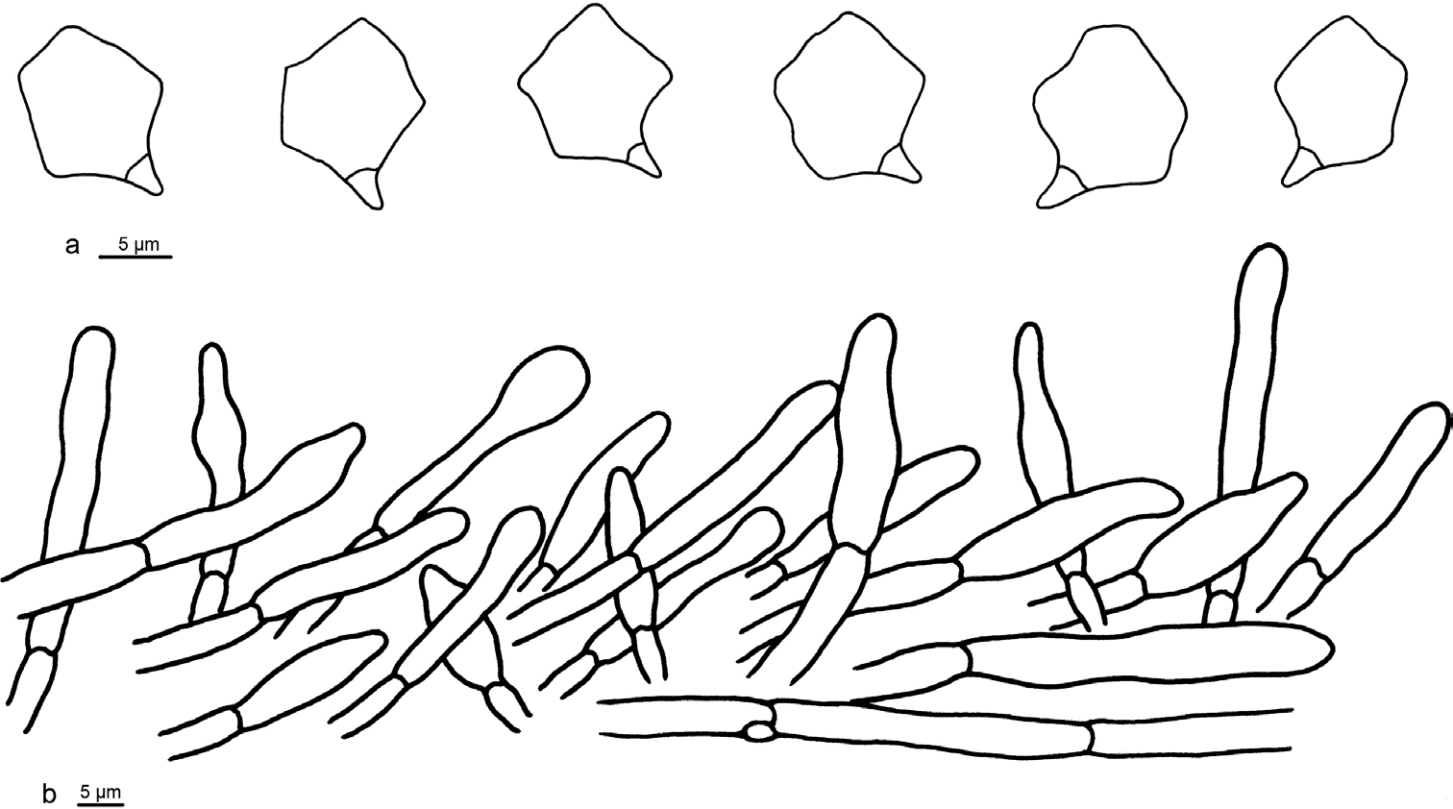
Microscopic structures of *Entoloma pleurotoides* (holotype): **a** Basidia **b** Basidiospores **c** Pileipellis.

#### 5. Entoloma reductum

Taxon classificationFungiAgaricalesEntolomataceae

Xiao L. He & E. Horak sp. nov.

2538DA48-2E22-5588-8DCB-CF6374E03CC6

817513

**Figure 6.**
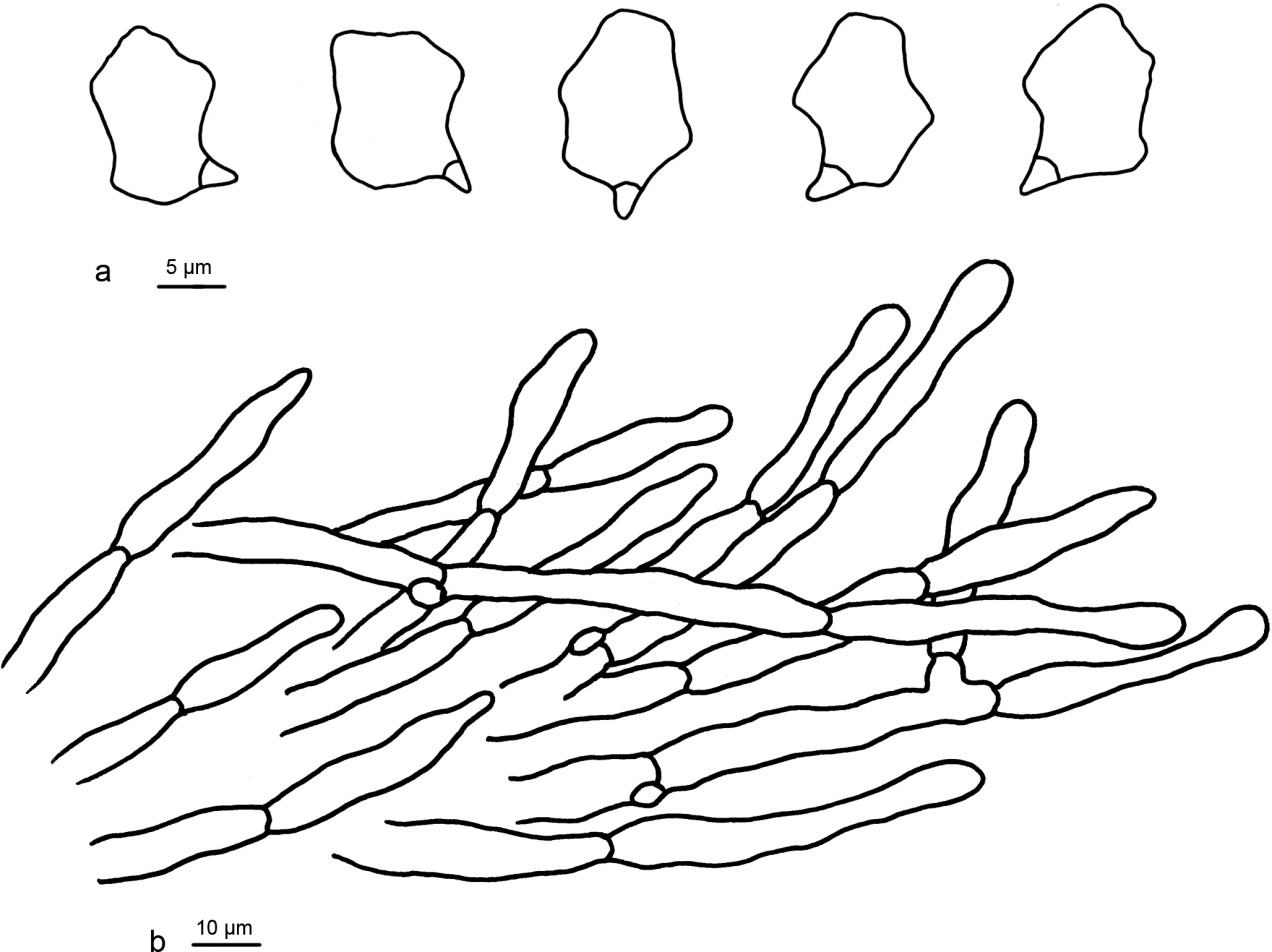
Microscopic structures of *Entoloma reductum* (holotype) **a** Basidiospores **b** Pileipellis.

Figures 1i, j, k , 6

##### Type.

China. Yunnan Prov.: Binchuan County, Jizu Mountain, ca. 2600 m elev., 25°58'N, 100°21'E, on rotten stump of *Castanopsis* sp., 8 September 2015, *X.L. He* (*SAAS 1091*, holotype; *ZT 13607*, isotype).

##### Sequences ex holotype.

KU312123 (ITS), KU534232 (nLSU), KU534480 (RPB2), KU534419 (mtSSU).

##### Etymology.

*reductum* (Lat.), referring to the reduced stipe.

##### Diagnosis.

*Entoloma reductum* is similar to *E. byssisedum* but differs by the size of the basidiospores.

Pileus 8–25 mm broad, conchate, broadly convex to applanate, greyish at first, becoming greyish-brown with age, entirely matted-tomentose or matted-fibrillose, fibrils greyish-white, slightly hygrophanous, margin weakly transparent-striate. Lamellae moderately close, with two tiers of lamellulae, adnate, ventricose, up to 4 mm wide, whitish or pale greyish at first, becoming pink or rust pinkish with age, entire edges concolorous. Stipe 1–2.5 × 0.5–1 mm, lateral, strongly reduced, pale grey brownish, covered with minute pale greyish fibrils, base with white mycelium. Rhizoids absent. Context thin, greyish, unchanging on exposure. Odour absent. Taste not distinctive.

Basidiospores 8–10.5 (12) × 6–7.5 µm (*x* = 8.8 ± 0.2 × 6.6 ± 0.3 µm), Q = 1.25–1.61, ***Q*** = 1.35 ± 0.05, 5–6-angled, heterodiametric in profile view. Basidia 20–34 × 8–11 µm, clavate, 4-spored, clampless. Lamellar edge fertile. Cheilocystidia, pleurocystidia and caulocystidia absent. Pileipellis a cutis composed of cylindric hyphae, terminal cells (25–) 40–65 × 5–7 µm, repent or slightly uplifted, cylindrical or slender clavate, non-gelatinised wall thin, smooth or minutely encrusted with slightly pale brown pigment. Oleiferous hyphae present in pileipellis. Clamp connections present in the pileipellis.

##### Habitat.

On decayed stump of *Castanopsis* sp.; on soil or rock amongst moss in forest dominated by *Quercus* sp.

##### Additional materials examined.

China. Sichuan Prov.: Yajiang County, Gexigou National Nature Reserve, ca. 2800 m elev., 30°03'N, 101°E, on rock amongst moss, 6 August 2015, *X.L. He* (*SAAS 1016*); on soil amongst moss, 3 August 2014, *X.L. He* (*SAAS 1897*); on rock amongst moss, 3 August 2014, *X.L. He* (*SAAS 2068*). Yunnan Prov.: Binchuan County, Jizu Mountain, ca. 2600 m elev., 25°58'N, 100°21'E, on decayed stump of *Castanopsis* sp., 8 September 2015, *X.L. He* (*SAAS 1608*; *ZT 13606*).

##### Remarks.

*Entoloma reductum* is unique by the combined features of pleurotoid, greyish-brown basidiomes, presence of brownish encrusting and intracellular pigment and presence of scattered clamp connections.

*Entoloma reductum* can be confused with *E. byssisedum* (Pers.) Donk; however, the latter species is separated by the larger basidiospores (9.5–12 × 6.5–8.0 µm, Noordeloos 2004). E. byssisedum var. microsporum is separated by the more reniform and paler coloured pileus and, in addition, the two species are well distinguished by their ITS and RPB2 sequences. *Claudopus dulcisaporus* Largent, described from North America, shares with *E. reductum* the brown basidiomes and the size of the basidiospores, but it can be distinguished not only by the presence of abundant cheilocystidia, but also the habitat (Largent 1994). *Claudopus graveolens* Largent is distinguished by smooth and bicolorous pileus, presence of cheilocystidia (Largent 1976); finally, *C. avellaneus* differs by the narrower basidiospores (8–10 × 5–6 µm, Murrill 1917).

#### 6. Entoloma byssisedum var. microsporum

Taxon classificationFungiAgaricalesEntolomataceae

Esteve-Rav. & Noordel.

B2AA2990-48FB-56A3-A3C6-2F396B4F8804

Figures 1l, m , 7

##### Description of Chinese material.

Pileus 5–20 mm, reniform, broadly convex, expanding to applanate, whitish-grey to greyish, entirely matted-tomentose to matted-appressed fibrillose, fibrils whitish, slightly hygrophanous, not striate. Lamellae with 2–3 tiers of lamellulae, adnexed, ventricose, up to 2.5 mm wide, moderately close, pale greyish at first, becoming greyish-pink, entire margin concolorous. Stipe 1–5 × 0.5–1 mm, strongly reduced, lateral, grey, covered with minutely, pale greyish fibrils, at base with white hairy mycelium. Basal rhizoids present, white. Context thin, unchanging. Odour absent. Taste not distinctive.

Basidiospores 8–10 × 5.5–7 (7.5) µm (*x* = 9 ± 0.3 × 6.5 ± 0.2 µm), Q = 1.29–1.52, ***Q*** = 1.39 ± 0.04, 5–6 (7)-angled, heterodiametric in profile view. Basidia 30–34 × 9–11 µm, clavate, 4-spored, rarely 2-spored, clampless. Lamellar edge fertile. Cheilocystidia, pleurocystidia and caulocystidia absent. Pileipellis a cutis composed of cylindrical hyphae, repent terminal cells (30–) 35–50 × 4–7 µm, cylindrical (or slender subclavate), non-gelatinised wall thin, smooth or minutely encrusted with pale brown pigment. Oleiferous hyphae numerous in pileipellis. Clamp-connections present in the pileipellis.

##### Habitat.

On decaying stump of *Betula* sp. in deciduous forest dominated by *Betula* sp. and *Quercus* sp.

##### Materials examined.

China. Sichuan Prov.: Kangding County, Mugecuo, ca. 2700 m elev., 30°13'N, 101°83'E, on decaying stump of *Betula* sp., 4 August 2015, *X.L. He* (*SAAS 1160*); on decaying stump of *Betula* sp., 3 September 2015, *X.L. He* (*SAAS 1828*); on decaying stump of *Betula* sp., 3 September 2015, *X.L. He* (*SAAS 1279*; *ZT 13608*). Xizang Autonomous Region (Tibet): Linzhi County, Kadinggou, ca. 2980 m elev., 29°50'N, 93°25'E, on decaying stump of *Betula* sp., 25 September 2014, *X.L. He* (*SAAS 1025*); Linzhi County, Sejila Mountain, ca. 3600 m elev., 29°35'N, 94°25'E, on decaying stump of *Betula* sp., 24 September 2014, *X.L. He* (*SAAS 1953*).

##### Remarks.

Entoloma byssisedum var. microsporum closely resembles typical *E. byssisedum* by its small crepidotoid pale greyish-brown basidiomes whose pileipellis is covered with fine, whitish arachnoid fibrils and lateral, strongly reduced to absent stipe. However, the basidiospores of the Chinese specimens are distinctly smaller as recorded for typical *E. byssisedum* and, thus, the morphotaxonomic characters correspond well with European collections of E. byssisedum var. microsporum (Noordeloos 2004). The identification is further supported by ITS sequences which demonstrate that the Chinese specimens of E. byssisedum var. microsporum are 99% identical as compared to those reported for European material (KJ001409). It is noteworthy that, in GenBank, there are two sequences labelled “*E. byssisedum*” (EU784209, KJ001413) which, however, are significantly different. The ITS sequences of the Chinese E. byssisedum var. microsporum are 97% identical to EU784209 but do not correspond with KJ001413. Accordingly, it could be speculated that E. byssisedum var. microsporum and E. byssisedum var. byssisedum actually represent two different species.

**Figure 7.**
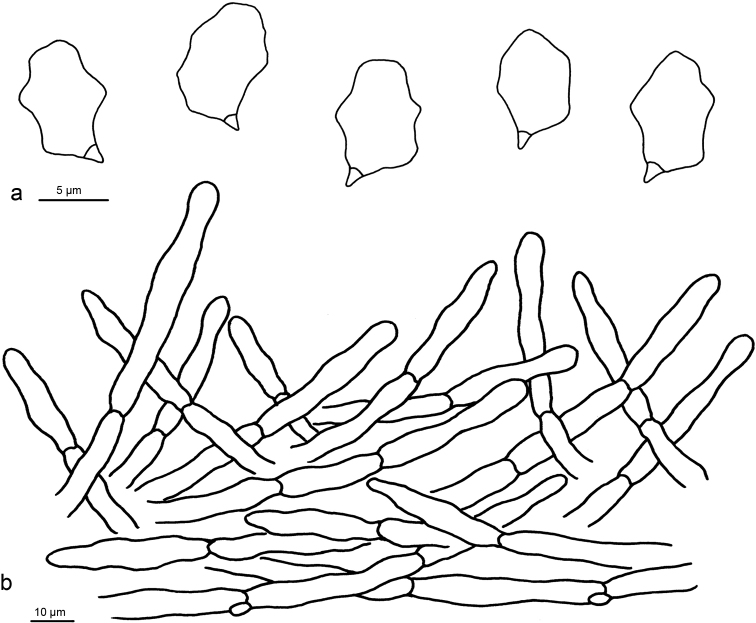
Microscopic structures of Entoloma byssisedum var. microsporum (SAAS 1279) **a** Basidiospores **b** Basidia **c** Pileipellis.

### Key to the Chinese species of Entoloma subgenus Claudopus with pleurotoid basidiomes

**Table:** 

1	Pileus grey to greyish-brown	**2**
–	Pileus white to pinkish-white	**3**
2	Basidiospores 8–9.5 (–10) × (5.5) 6–7 µm. Occurring on bark-wood of *Castanopsis* and/or on soil	***E. reductum***
–	Basidiospores 9–10 × 6–6.5 µm. Occurring on decayed stumps of *Betula*	**Entoloma byssisedum var. microsporum**
3	Basidiospores 8.5–10 (–10.5) × 7.5–9 µm, subisodiameteric to isodiameteric	***E. pleurotoides***
–	Basidiospores smaller, heterodiameteric	**4**
4	Reported from tropical lowland rain forest. Basidiospores 8–9 × 6–7 µm. On soil	***E. crepidotoides***
–	Reported from xerophytic or from montane habitat	**5**
5	Basidiospores 8–10 × 6.5–7.5 µm. Basidiomes white at first becoming yellowish to orange with age. On living stem, on fallen branches of conifers or on soil	***E. conchatum***
–	Basidiospores narrower, on living or decaying hardwoods	**6**
6	Basidiomes white at first becoming yellowish to orange with age. On decaying debris of fagalean tree.	***E. flabellatum***
–	Basidiomes persistently white. Amongst moss at base of living *Castanopsis*	***E. gregarium***

### *Molecular analyses*

**Figure 8.**
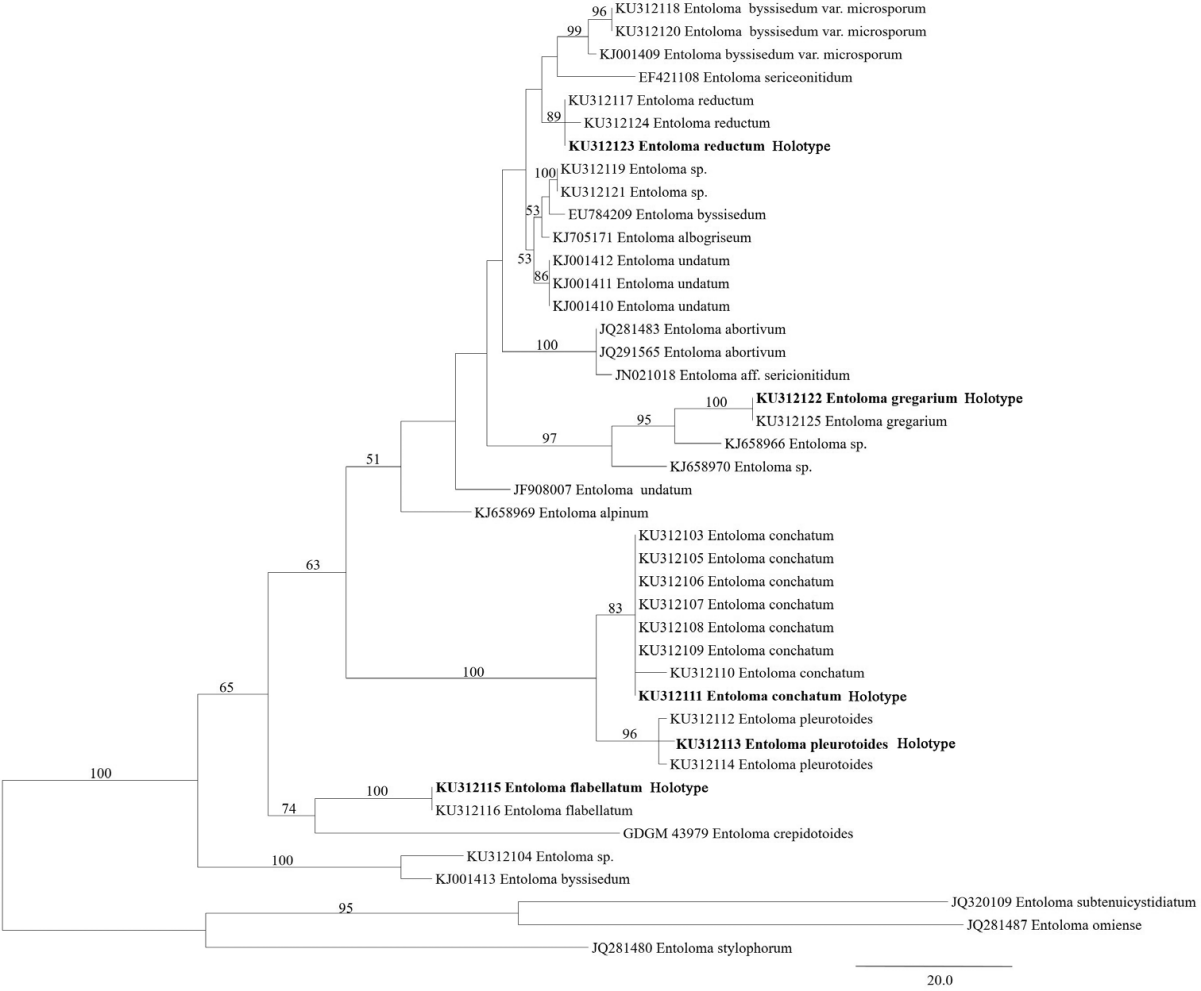
Phylogenetic reconstruction of *Claudopus* based on ITS sequences. Maximum parsimony bootstrap values (BS > 50%) are indicated above or below the branches, new species are in bold.

The new sequences presented in this study are deposited in GenBank with accession numbers KU312103–KU312125, KU534215–KU534217, KU534219–KU534238, KU534415–KU534436 and KU534459–KU534482. In the phylogenetic analysis, the final ITS dataset included 43 sequences; *E. omiense*, *E. stylophorum* and *E. subtenuicystidiatum* were designed as outgroups. The ITS dataset contained 702 nucleotide sites, of which 416 characters were constant, 175 were parsimony-informative characters and 111 variable characters were parsimony-uninformative. Two most parsimonious trees were recovered, based on ITS sequences and one of them is shown (Fig. 8). In the ITS tree, *E. conchatum* and *E. pleurotoides* grouped in the same monophyletic clade, while the remaining three species are placed in three different clades.

**Figure 9.**
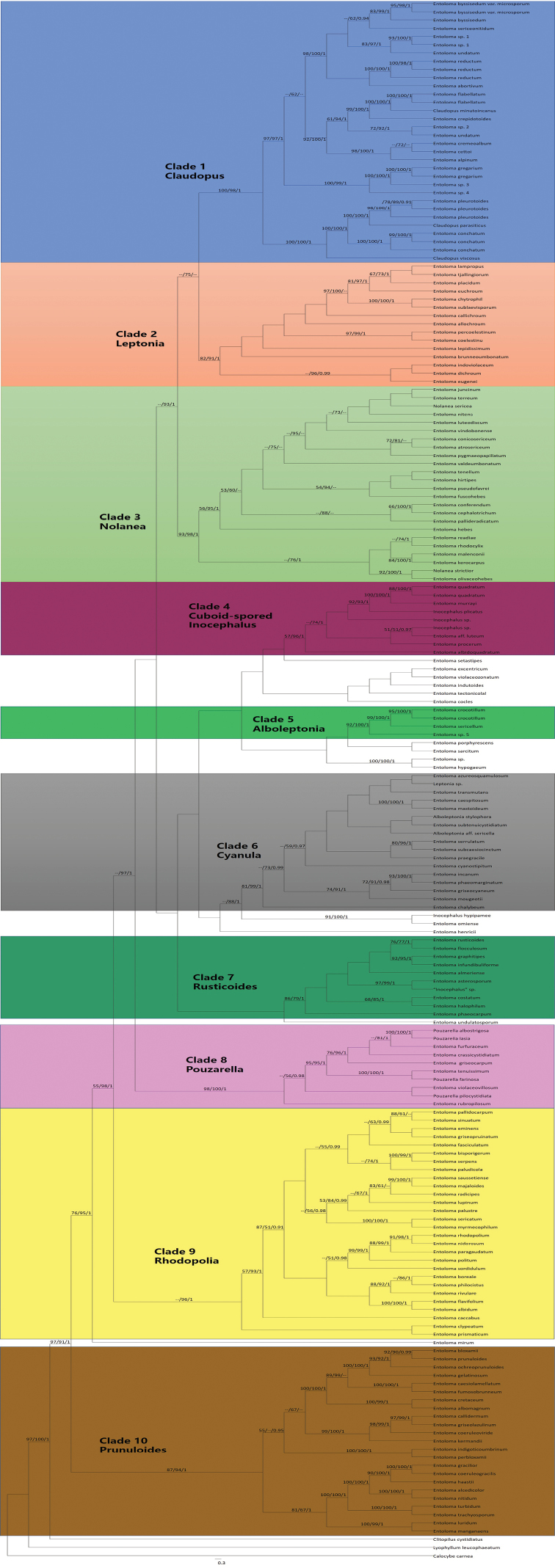
Cladogram based on the combined ITS, LSU, RPB2 and mtSSU sequences resulting from ML analysis. New species of Entoloma subgenus Claudopus are in bold. MPBS support values (> 50%), RAxML BS support values (> 50%) and Bayesian posterior probability values (BPP> 0.90) are indicated above or below branches as MPBS/RAxML BS/BPP.

The combined dataset (ITS, nLSU, mtSSU and RPB2) consisted of 190 representatives and 4028 nucleotide bases were included. *Calocybe carnea* (Bull.) Donk, *Clitopilus cystidiatus* Hauskn. & Noordel. and *Lyophyllum leucophaeatum* (P. Karst.) P. Karst. were selected as outgroups. MP, ML and Bayesian analyses produced similar typologies except for some unsupported clades and the cladogram resulting from ML analysis is shown (Fig. 9). Ten monophyletic clades viz. *Claudopus* (Clade 1), *Leptonia* (Clade 2), *Nolanea* (Clade 3), *Cuboid-spored Inocephalus* (Clade 4), “*Alboleptonia*” (Clade 5), *Cyanula* (Clade 6), *Rusticoides* (Clade 7), *Pouzarella* (Clade 8), *Rhodopolia* (Clade 9) and *Prunnuloides* (Clade 10) were observed in the analyses. In Bayesian and ML analyses, *Nolanea*, *Leptonia* and *Claudopus* grouped in a large clade with significant support (0.99 pp and 93 RAxML BS, respectively). In the *Claudopus* clade, the five new species described above are clearly separated from each other. The traditional *Inocephalus* species (*E. griseolazulinum* Manim. & Noordel., *E. indigoticoumbrinum* G.M. Gates & Noordel., *E. tectonicola* Manim. & Noordel., *Inocephalus hypipamee* Largent and the cuboid-spored *Inocephalus*) are not placed in the same clade, and so for the sequestrate *Entoloma* (*E. prismaticum* Hir. Sasaki, A. Kinosh. & K. Nara, *E. hypogaeum* Hir. Sasaki, A. Kinosh. & K. Nara, *E. asterosporum* (Coker & Couch) Noordel. & Co-David and *Entoloma* sp.).

## Discussion

Five new species of Entoloma subgenus Claudopus from China were described based on morphological and molecular data. Additionally, the phylogeny of *Entoloma* was also carried out, based on the combined ITS, LSU, mtSSU and RPB2 sequences. Co-David et al. (2009) presented a relatively comprehensive molecular phylogeny on Entolomataceae (54 representatives of *Entoloma* were included). Since then, several molecular studies focusing on entolomatoid agarics have been reported (Baroni and Matheny 2011; Baroni et al. 2011; He et al. 2013; Kinoshita et al. 2012; Kokkonen 2015; Morgado et al. 2013; Morozova et al. 2014; Vila et al. 2014). Most of these studies were focused on one certain group or subgenus in *Entoloma* s.l. and samples used in phylogenetic analyses were mainly limited to the studied groups (He et al. 2013; Kokkonen 2015; Morgado et al. 2013; Morozova et al. 2014; Vila et al. 2014). This study provides a more comprehensive phylogeny, based on the sequences newly generated and includes almost all the representatives published in those previous studies (187 representatives of *Entoloma* were included). This is also the first contribution providing a relatively densely sampled systematic treatment of *Claudopus*. It is noteworthy that *Claudopus* in a traditional morphological sense is not monophyletic and the Rusticoides-group, previously considered within *Claudopus*, formed a separate clade; but the pleurotoid or crepidotoid *Claudopus* members, as well as *E. abortivum* (Berk. & Curt.) Donk, *E. undatum* and its related taxa, are placed in a distinctive monophyletic clade.

### Phylogenetic analyses of combined datasets

In combined phylogenies, *Entoloma* s.l. is divided into ten monophyletic groups (Fig. 9). The majority of these groups are well defined regarding macro or micro-morphology. The most distinctive group is the *Pouzarella*, whose taxonomic position as a monophyletic group is also supported by both morphologic and molecular evidence (Baroni and Matheny 2011; Co-David et al. 2009; He et al. 2013). In the previous studies, *Claudopus* species were nested in the nolanea-claudopus clade, based on very limited samples (Baroni and Matheny 2010; Co-David et al. 2009). However, on the basis of a more comprehensive sampling in the present study, it is obvious that *Claudopus* (*E. rusticoides* and relatives are excluded) is a monophyletic group clearly separated from *Nolanea*. *Claudopus* and *Nolanea* species are grouped as monophyletic groups, respectively, in different clades. In the present analysis, clade *Rusticoides* (traditionally considered within *Claudopus*) is also recognised to be monophyletic and is separated from *Claudopus*. Research data have indicated that traditional *Leptonia* (either as genus or subgenus) might be polyphyletic and species accommodated in section Cyanula are distantly related to section Leptonia (Baroni and Matheny 2011; Co-David et al. 2009). In Noordeloos and Gates (2012), section Cyanula was raised to subgeneric level and the concept of subgenus Leptonia was also emended. The present analysis further proved that the traditional concept of subgenus Leptonia is polyphyletic. In the previous studies, section Leptonia belonged to the /nolanea-claudopus clade, section Cyanula to the /inocephalus-cyanula clade, respectively. However, monophyly of section Leptonia and Cyanula are clearly demonstrated in this study. Section Leptonia is clearly separated from *Nolanea* and *Claudopus* and *Cyanula* is distinguished from *Inocephalus* (cuboid-spored *Inocephalus*), respectively. Two taxa in the traditional “*Alboleptonia*” (*A. stylophora* and A. aff. sericella) are grouped in the monophyletic clade *Cyanula*, while the following species of “*Alboleptonia*” (*Entoloma crocotillum* Xiao L. He and *E. sericellum* (Fr.) P. Kumm.) are placed in a separated clade. These results suggest that “*Alboleptonia*” in the traditional circumscription might not be a well-defined genus or subgenus and the concept of *Cyanula* also needs to be revised. Polyphyly of the traditional *Inocephalus* is further confirmed in the present analyses. However, all analysed cuboid-spored species belonging to *Inocephalus* are clustered in a monophyletic clade, suggesting that these species may represent a distinct monophyletic group. The clade *Rhodopolia* (Clade 9) received strong support in the ML and Bayesian analysis which, however, is not supported by MP analysis. In the strongly supported Prunuloides clade, members in the emended subgenus Entoloma (Noordeloos and Gates 2012) were included and it is distant from the subgenus Rhodopolia, which is consistent with the previous studies (Baroni and Matheny 2011; Co-David et al. 2009). One internal clade includes 5 species of the traditional *Prunuloides* and 4 species [*E. haastii* G. Stev., *E. nitidum* Quél., *E. trachyosporum* Largent and *E. turbidum* (Fr.) Quél.] that belong to the genus *Entocybe* established by Baroni et al. (2011); however, it seems that it will cause subgenus Prunuloides to be paraphyletic if these entocyboid species are excluded from subgenus Prunuloides for the time being. The results also showed that sequestrate form (*E. prismaticum*, *E. hypogaeum*, *E. asterosporum* and *Entoloma* sp.) in *Entoloma* have multiple origins, which is consistent with the previous studies (Baroni and Matheny 2011; Kinoshita et al. 2012).

In conclusion: It can be safely predicted that, within entolomatoid agarics, further monophyletic lineages will be discovered as soon as the number of molecular information increases and, subsequently, the present classification of *Entoloma* s.l. is going to change fundamentally.

### Phylogenetic species determination of *Claudopus*

The variation of macroscopic characters in many species of Entoloma subgenus Claudopus is limited and, accordingly, it is difficult or even impossible to identify morphologically similar taxa. In the future, it will be essential to recognise and describe new species of Entoloma subgenus Claudopus in combination with molecular data and morphological characters. Molecular analyses convincingly show that, regarding species of *Claudopus*, both ITS and RPB2 sequences have a high discriminating potential to separate species. In the present phylogenetic data, thirty-five sequences representing twenty-one phylogenetic species of Entoloma subgenus Claudopus are uncovered. It is herewith emphasised that nine species have now been recorded and four unidentified species have been collected from China which suggests that species diversity of Entoloma subgenus Claudopus is high in this country.

In the present ITS analysis (Fig. 8), two sequences labelled “*Entoloma byssisedum*” (EU784209, KJ001413) and one sequence referred to as E. byssisedum var. microsporum (KJ001409) are included. The relevant voucher material was collected in Europe. However, the molecular tree(s) demonstrates that the three sequences are nested in different clades: KJ001413 is distant from EU784209 and KJ001413. Consequently, it can be speculated that the European E. byssisedum var. microsporum could be a distinctive species rather than a variety. Furthermore, the sequenced European materials, identified as *E. byssisedum*, is composed of a complex of as yet unnamed species or even more likely to misidentification of species of similar basidiomes. A similar situation is observed regarding *E. undatum*. In GenBank, sequences retrieved from several collections of *E. undatum* (ITS: KJ001412, JF908007; LSU: AF207199, GQ289202, KJ001455) are obviously different and belong to different species taxonomically not disentangled yet.

### Ecology of Chinese taxa of *Claudopus*

Fieldwork demonstrated that the Chinese species of *Claudopus* are found in different habitats, characterised by distinctive ecology and substrates, (e.g. bark-wood of decaying debris of fallen or live broadleaf trees and conifers, soil in grassland or moss-covered rock). Unlike other entolomatoid species, the habitat on above-ground decaying wooden substrates is the rule for members of Entoloma subgenus Claudopus, while occurrence on bark-wood of live trees was not mentioned before in the relevant literature. The present results indicate, however, that growth on bark-wood of live trees is not uncommon for Chinese species of Entoloma subgenus Claudopus.

Based on present records, it is remarkable that, in China, the distribution of *Claudopus* stretches from tropical lowland forests (*E. crepidotoides*, from Hainan Prov.) to temperate and finally also to alpine localities in Sichuan Prov. (*E. alpinum*) or Xizang Autonomous Region, Tibet (E. byssisedum var. microsporum).

It must be emphasised that ecological data alone do not help to identify the Chinese taxa reported in the present contribution. As an example: based on available reports, *E. reductum* was found in different habitats and localities. The records from Sichuan Prov. were discovered on soil and moss-covered rock, but the specimens gathered in Yunnan Prov. are lignicolous. Despite different habitats, the microscopic examination and molecular data confirmed that the aforementioned records of *E. reductum* are identical. A similar observation was made regarding *E. conchatum* and *E. pleurotoides*. To confirm the identification of *Claudopus*, based on references relating to substrate/habitat, are not reliable unless morphotaxonomic characters and molecular data are also taken into consideration.

### Diagnostic characters of *Claudopus* and the taxonomic position of *Entoloma*abortivum

In the large majority of entolomatoid species, the stipe is central and well developed. However, in some taxa of Entoloma subgenus Claudopus, the stipe is absent or strongly reduced and some species possess well-developed but eccentrically inserted stipes. In the tested *Claudopus* species, viz. *E. abortivum*, *E. alpinum*, *E. undatum*, *E. sericeonitidum* (P.D. Orton) Arnolds and an unidentified taxon (SAAS 1154), the stipes are well developed; while in other taxa strongly reduced and laterally attached stipes are observed. All of the above enumerated species are nested in the *Claudopus* clade with a strong support. In the study of Vila et al. (2014), *E. korhonenii* Noordel., as well as several other *Claudopus* species, form a monophyletic group based on ITS sequences. The only common macro-character derived from the descriptions of these members, except for *E. undatum*, *E. korhonenii* and *E. sericeonitidum*, is that the stipes are off-centre which might be a key character for the recognition of *Claudopus*. It should be emphasised that the eccentric stipe should be a stabile and reliable character rather than an accidental adaptation to substrate in the habitats. During our field survey, we always found that the stipes of *E. abortivum*, *E. alpinum* and SAAS 1154 are off-centre or even eccentric.

Apparently, the stipe is also eccentrically inserted - but not properly mentioned in the relevant descriptions - in basidiomes of *E. undatum*, *E. sericeonitidum* and *E. korhoneni*. The eccentric position of the stipe can be observed in photographs of *E. undatum* and *E. korhonenii* (Noordeloos 2004). The picture of *E. sericeonitidum* (*Paraeccilia sericeonitida*) in Largent (1994) also showed that the stipe is slightly off-centre. In the future, the character “position of stipe” should be taken more carefully into consideration to support the hypothesis that this diagnostic feature is characteristic for the delimitation of Entoloma subgenus Claudopus.

Based on published records and collections from China, basidiomes of *E. abortivum* occur in two morphs viz. first with typical agaricoid and second with secotioid basidiomes. Regardless ofthe insertion of the stipe, the taxonomic position of *E. abortivum* was uncertain until molecular methods revealed that this taxon actually represents a typical species of Entoloma subgenus Claudopus (Baroni and Matheny 2011; Co-David et al. 2009; He et al. 2013; Vila et al. 2014). In the current descriptions of *E. abortivum*, the attachment of the stipe is not mentioned in particular. However, in our Chinese material, the position of the stipe of *E. abortivum* is always slightly but distinctly off-centre.

## Supplementary Material

XML Treatment for Entoloma conchatum

XML Treatment for Entoloma flabellatum

XML Treatment for Entoloma gregarium

XML Treatment for Entoloma pleurotoides

XML Treatment for Entoloma reductum

XML Treatment for Entoloma byssisedum var. microsporum
