# Corrigendum: He XL, Horak E, Wang D, Li TH, Peng WH, Gan BC (2019) Descriptions of five new species in Entoloma subgenus Claudopus from China, with molecular phylogeny of *Entoloma* s.l. MycoKeys 61: 1–26. https://doi.org/10.3897/mycokeys.61.46446

**DOI:** 10.3897/mycokeys.63.49739

**Published:** 2020-03-02

**Authors:** Xiao-Lan He, Egon Horak, Di Wang, Tai-Hui Li, Wei-Hong Peng, Bing-Cheng Gan

**Affiliations:** 1 Soil and Fertilizer Institute, Sichuan Academy of Agricultural Sciences, Chengdu 610066, China Soil and Fertilizer Institute, Sichuan Academy of Agricultural Sciences Chengdu China; 2 Schlossfeld 17, A-6020 Innsbruck, Austria Unaffiliated Innsbruck Austria; 3 State Key Laboratory of Applied Microbiology Southern China, Guangdong Provincial Key Laboratory of Microbial Culture Collection and Application, Guangdong Institute of Microbiology, Guangdong Academy of Sciences, Guangzhou 510070, China Guangdong Institute of Microbiology, Guangdong Academy of Sciences Guangzhou China

## Abstract

Entoloma subgenus Claudopus is widely distributed, yet the taxonomy and systematics of its species are still poorly documented. In the present study, more than forty collections of Claudopus were gathered in China and subsequently analysed, based on morphological and molecular data. The results revealed first a high level of species diversity of Claudopus in China and second, there is a wide ecological range regarding the substrates and the habitats ranging from temperate, tropical to subalpine locations. Based on morphological and molecular evidence, five novel species from China are proposed, viz. E. conchatum, E. flabellatum, E. gregarium, E. pleurotoides and E. reductum. Molecular phylogeny of Entoloma s.l. was also reconstructed, based on 187 representatives of Entoloma s.l. by employing the combined ITS, LSU, mtSSU and RPB2 sequences. Ten monophyletic clades (Claudopus, Leptonia, Nolanea, Cuboid-spored Inocephalus, &ldquo;Alboleptonia&rdquo;, Cyanula, Pouzarella, Rhodopolia, Prunuloides and Rusticoides) were recovered, while 13 taxa could not be placed in any defined clades. The results confirmed that Claudopus in a traditional morphological sense is not monophyletic and the Rusticoides-group, previously considered within Claudopus, formed a separate clade; but section Claudopus and relatives of E. undatum belong to a distinctive monophyletic group. Despite some monophyletic groups in Entoloma s.l. being distinctive in both morphology and molecular phylogeny, they were still treated as subgenera of Entoloma s.l. temporarily, because accepting them as genera will make Entoloma s.l. paraphyletic.

We found that Table 1 “A list of taxa, specimens and GenBank accession numbers of sequences used in this study” had been omitted and figure legends 1–7 were not the final version after our manuscript was published. Table 1 and revised figure legends 1–7 are now provided below.

**Table 1.** A list of taxa, specimens and GenBank accession numbers of sequences used in this study.

Taxa	Collection No.	Origin	GenBank accessions			
			ITS	LSU	RPB2	mtSSU
Alboleptonia aff. sericella	MCA1978	—	—	GU384609	GU384632	GU384583
*A. stylophora*	AST84	—	—	GU384610	GU384633	GU384584
*Calocybe carnea*	—	—	AF357028	AF223178	DQ367432	AF357097
*Claudopus minutoincanus*	DLL 9871	Australia	—	HQ731514	HQ731517	HQ731511
*C. viscosus*	DLL 9788	Australia	—	HQ731516	HQ731518	HQ731513
*Clitopilus hirneolus*	MEN 199956	Italy	—	GQ289211	GQ289278	GQ289352
*E. abortivum*	H den Bakker 92	Canada	—	GQ289150	GQ289222	GQ289290
*E. albidoquadratum*	P. Manimohan 667, holotype	India: Kerala	—	GQ289150	GQ289222	GQ289290
*E. albidum*	YL 3218	Canada	KC710102	KC710151	—	KC710180
*E. albomagnum*	Gates E2030	Australia	KC710065	KC710137	—	KC710165
*E. alcedicolor*	E. Arnolds 02-760276, holotype	The Netherlands	KC710123	GQ289152	GQ289224	GQ289292
*E. allochroum*	JVG 1060902-1	Spain	KC898376	KC898522	—	KC898488
*E. almeriense*	LIP JVG 990105I, holotype	Spain: Almer A	KJ001428	—	—	—
*E. alpinum*	SAAS 774, holotype	China: Sichuan	KJ658969	KJ658972	—	—
*E. asterosporum*	K479	Japan: Okayama	AB691990	AB692007	AB692017	—
*E. atrosericeum*	K69-310 G262084, holotype	France	LN850607	—	—	—
*E. azureosquamulosum*	GDGM 27355	China: Guangdong	JQ410333	JQ410325	—	JQ993073
*E. bisporigerum*	KK 106/02	Finland	LN850534	—	LN850682	—
*E. bloxamii*	MEN 200442	Austria, EU	KC710087	GQ289154	GQ289226	GQ289294
*E. brunneoumbonatum*	CAL 317, holotype	India	KX774266	—	—	—
*E. boreale*	KK 106/09, holotype	Finland	LN850624	—	LN850697	—
E. byssisedum var. microsporum	SAAS 1160	China: Sichuan	**KU312118**	**KU534231**	**KU534476**	**KU534421**
E. byssisedum var. microsporum	SAAS 1828	China: Sichuan	**KU312120**	**KU534234**	**KU534477**	**KU534433**
*E. caccabus*	MEN 200324	Belgium	KC710063	GQ289155	GQ289227	GQ289295
*E. caesiolamellatum*	Wölfel, 20.2.2000, holotype	Canary Islands	KC710126	KC710157	—	KC710187
*E. caespitosum*	GDGM 27564	China: Hainan	JQ281477	JQ320130	JQ993078	JQ993070
E. callichroum var. venustum	G. Wölfel, F. Hampe (L, Wö E17/10, holotype *E. venustum*)	Germany	KC898355	KC898523	—	KC898490
*E. callidermum*	Stubbe 06252 (GENT)	The Netherlands	KC710115	KC710153	—	KC710183
*E. cephalotrichum*	C. Ulje 1997-08-01 Netherlands	The Netherlands	—	GQ289157	GQ289229	GQ289297
*E. cettoi*	Zuccherelli et al. 11.IX.1992, holotype	Italy	LN850560	—	LN850687	—
*E. chalybeum*	E. Morozova (LE254353)	Russia: Leningrad	KC898445	KC898500	—	KC898465
*E. chytrophilum*	R.M. Dähncke (L 855, holotype)	Spain: Canary Islands	KC898434	KC898519	—	KC898479
*E. clypeatum*	MEN 198302	The Netherlands	KC710059	KC710136	—	KC710164
*E. cocles*	J. Vauras 9770F	Finland	—	GQ289230	GQ289159	GQ289299
*E. coelestinum*	L. Marina (LE258103)	Russia: Sverdlovsk	KC898362	KC898524	—	KC898494
*E. coeruleogracilis*	MEN 2004055	Australia: Tasmania	KC710107	GQ289167	GQ289238	GQ289307
*E. coeruleoviride*	Stubbe 06236	Malaysia	KC710057	KC710134	—	KC710162
*E. conchatum*	SAAS 1117	China: Sichuan	**KU312103**	**KU534225**	**KU534463**	**KU534420**
*E. conchatum*	SAAS 1014	China: Sichuan	**KU312105**	**KU534224**	**KU534462**	**KU534418**
*E. conchatum*	SAAS 1712, holotype	China: Sichuan	**KU312111**	**KU534220**	**KU534459**	**KU534432**
*E. conferendum*	MEN 200330	Slovakia	KC710055	KC710133	KC710191	KC710161
*E. conicosericeum*	LIP JVG 1080514, holotype	—	JX454878	—	—	—
*E. costatum*	G. Immerzeel 2000-10-10	The Netherlands	—	GQ289161	GQ289232	GQ289301
*E. crassicystidiatum*	GDGM 28821, paratype	China: Guangdong	KC678997	JQ291567	JQ993085	JQ993058
*E. cremeoalbum*	O300037, holotype	Norway	LN850559	—	LN850686	—
*E. crepidotoides*	GDGM 43979, holotype	China: Hainan	KJ958982	KJ958983	KJ958984	KJ958985
*E. crepidotoides*	GDGM 29287	China: Hainan	—	KM581267	KM581269	KM581268
*E. cretaceum*	G. Gates E 1181, holotype	Australia: Tasmania	KC710064	GQ289162	GQ289233	GQ289302
*E. crocotillum*	SAAS 255, holotype	China: Sichuan	KC555561	KC555558	KP226185	—
*E. crocotillum*	SAAS 175	China: Sichuan	KC555560	KC555557	—	—
*E. cyanostipitum*	GDGM 31318, holotype	China: Jilin	NR154977	KY972694	—	—
*E. dichroum*	LE234260	Russia: Zhiguli	KC898442	KC898528	—	KC898487
*E. eminens*	KK 417/12, holotype	Finland	LN850584	—	—	—
*E. euchroum*	LE262995	Russia: Caucasus	KC898417	KC898516	—	KC898483
*E. eugenei*	E. Popov (LE253771 holotype)	Russia: Primorsky	KC898438	KC898529	—	—
*E. excentricum*	M. Meusers E 1705	Germany	—	GQ289163	GQ289234	GQ289303
*E. fasciculatum*	L.R. Hesler 29376, holotype	U.S.A.	LN850614	—	—	—
*E. flabellatum*	SAAS 1501	China: Guizhou	**KU312115**	**KU534215**	**KU534471**	—
*E. flabellatum*	SAAS 1080, holotype	China: Guizhou	**KU312116**	**KU534217**	**KU534470**	—
*E. flavifolium*	Y. Lamoureux 2846 (CMMF)	Canada, Québec	KC710097	KC710150	—	KC710179
*E. flocculosum*	JVG 1080920-20	Spain: Barcelona	KJ001438	KJ001463	—	—
*E. fumosobrunneum*	MEN2005120, holotype	Canada, Newfoundland	KC710125	KC710156	—	KC710186
*E. furfuraceum*	GDGM 28818, holotype	China: Jinlin	JX975293	JQ993094	JQ993084	JQ993062
*E. fuscohebes*	LIP JVG 960127, holotype	—	JX454908	—	—	—
*E. gelatinosum*	G. Gates E792	Australia: Tasmania	KC710103	GQ289165	GQ289236	GQ289305
*E. gracilior*	G. Gates E1220	Australia: Tasmania	KC710112	GQ289169	GQ289240	GQ289309
*E. graphitipes*	JVG 1071208-10	Spain: Bizkaia	KJ001449	KJ001458	—	—
*E. gregarium*	SAAS 1220, holotype	China: Yunnan	**KU312122**	**KU534237**	**KU534474**	**KU534423**
*E. gregarium*	SAAS 1493	China: Yunnan	**KU312125**	**KU534238**	**KU534475**	**KU534430**
*E. griseocarpum*	SAAS 1230, holotype	China: Tibet	MH020753	KU534253	KU534500	KU534438
*E. griseocyaneum*	O. Morozova (LE254351)	Russia: Caucasus	KC898444	KC898498	—	KC898463
*E. griseolazulinum*	P. Manimohan 738, holotype	India: Kerala	—	GQ289166	GQ289237	GQ289306
*E. griseopruinatum*	JLC030924-8, isotype	France	LN850556	—	—	—
*E. haastii*	ME Noordeloos 2004055	Australia: Tasmania	—	GQ289167	GQ289238	GQ289307
*E. halophilum*	LIP JVG 961228H, holotype	Spain: Almer A	KJ001441	KJ001461	—	—
*E. hebes*	E. Hartman 1992-10-28	Netherlands	—	GQ289170	GQ289241	GQ289310
*E. henricii*	HKAS 63414	China: Henan	—	JQ410332	JQ993076	JQ993069
*E. hirtipes*	JVG 990510-2	—	JX454935	—	—	—
*E. hypogaeum*	K382, holotype	Japan: Oita	AB692001	AB692009	AB692019	—
*E. incanum*	HKAS 54614	China: Yunnan	JQ281488	JQ320127	—	—
*E. indigoticoumbrinum*	ME Noordeloos 200406 3, holotype	Australia: Tasmania	—	GQ289242	GQ289171	GQ289311
*E. indoviolaceum*	P. Manimohan 700, holotype	India: Kerala	—	GQ289172	GQ289243	GQ289312
*E. indutoides*	O. Morozova (LE254354)	Russia: Leningrad	KC898451	KC898503	—	KC898468
*E. infundibuliforme*	TENN:013964, holotype	USA: Tennessee	HQ179671	HQ179671	—	—
*E. juncinum*	JC-19981012.5a (Ex-1004)	—	JX454902	—	—	—
*E. kermandii*	G. Gates E227, holotype	Australia: Tasmania	—	GQ289173	GQ289244	GQ289313
*E. kerocarpus*	WU18878, holotype	Austria	LN850576	—	LN850688	—
*E. lampropus*	UPS: BOT: F-176490, neotype	Sweden	KC898377	KC898471	—	KC898506
*E. aff. Luteum*	GDGM 28991	China	—	JQ993093	JQ993075	—
*E. lepidissimum*	E. Popov (LE254871)	Russia: Novgorod	KC898363	KC898531	—	KC898493
*E. lupinum*	KK 13/14 & J. Vauras, holotype	Finland	LN850570	—	LN850695	—
*E. luteodiscum*	CAL 132, holotype	India	KX774267	—	—	—
*E. luridum*	MEN 2005108	Italy	KC710091	KC710146	KC710192	KC710175
*E. majaloides*	KK 782/12	Finland	LN850478	—	LN850654	—
*E. malenconii*	JVG 1111118-1	—	JX454946	—	—	—
*E. manganaense*	G. Gates E369, isotype	Australia: Tasmania	KC710085	KC710143	—	KC710172
*E. mastoideum*	GDGM 26597, holotype	China: Guangdong	JQ291564	JQ320126	—	—
*E. mirum*	KK 99/14, holotype	Finland	LN850548	—	LN850699	—
*E. mougeotii*	LE254352	Russia: Caucasus	KC898446	KC898499	—	KC898464
*E. murrayi*	QI 1001	China: Liaoning	KJ658967	JQ993090	JQ993081	JQ993064
*E. myrmecophilum*	G. Tjallingii-Beukers 1981-10-30	Netherlands	KC710120	GQ289174	GQ289245	GQ289314
*E. nidorosum*	KK 419/12	Finland	LN850503	LN850706	LN850673	—
*E. nitens*	JC-19981012.5b (Ex-1004)	—	JX454901	—	—	—
*E. nitidum*	ME Noordeloos 200426	Slovakia	KC710122	GQ289175	GQ289246	GQ289315
*E. ochreoprunuloides*	E. Arnolds 01-142, holotype	Germany	KC710092	KC710147	—	KC710176
*E. olivaceohebes*	Dhancke 2507	—	JX454932	—	—	—
*E. omiense*	GDGM 27563	China:	JQ281487	JQ410330	JQ993079	JQ993067
*E. pallideradicatum*	A. Hausknecht, isotype ex WU 189010	Austria	—	GQ289176	GQ289247	GQ289316
*E. pallidocarpum*	GDGM 28828	China: Jilin	JQ320106	JQ410331	JQ993080	JQ993074
*E. paludicola*	KK 386/12	Finland	LN850516	—	LN850678	—
*E. palustre*	KK 101/14, holotype	Finland	LN850592	—	LN850692	—
*E. parasiticum*	ME Noordeloos 200330	Belgium	—	GQ289177	GQ289248	GQ289317
*E. paragaudatum*	KK 383/08, holotype	Finland	LN850530	—	LN850691	—
*E. perbloxamii*	MEN 2004071, holotype	Australia: Tasmania	KC710117	GQ289178	GQ289249	GQ289318
*E. percoelestinum*	T. Bulyonkova (LE254327)	Russia: Novosibirsk	KC898359	KC898526	—	KC898496
*E. phaeocarpum*	LIP JVG 1031018, holotype	Spain: La Rioja	KJ001430	KJ001462	—	—
*E. phaeomarginatum*	ME Noordeloos 2004127	Australia: Tasmania	—	GQ289179	GQ289250	GQ289319
*E. philocistus*	Hausknecht & Reinwald 9.XI.1998, paratype	Portugal	LN850600	—	—	—
*E. placidum*	S. Lundell (5276) & G. Haglund (UPS: BOT: F-121714, epitype)	Sweden	KC898394	KC898514	—	KC898481
*E. pleurotoides*	SAAS 1215	China: Yunnan	**KU312112**	**KU534229**	**KU534467**	**KU534422**
*E. pleurotoides*	SAAS 1252, holotype	China: Yunnan	**KU312113**	**KU534227**	**KU534468**	**KU534424**
*E. pleurotoides*	SAAS 1354	China: Yunnan	**KU312114**	**KU534228**	**KU534469**	**KU534425**
*E. politum*	KK 289/09	Finland	LN850511	—	LN850677	—
*E. porphyrescens*	ME Noordeloos 2004113	Australia: Tasmania	—	GQ289182	GQ289253	GQ289322
*E. praegracile*	GDGM 29251	China: Guangdong	JQ281482	JQ320129	JQ993077	JQ993072
*E. prismaticum*	K381	Japan: Tokyo	AB691998	AB692006	AB692016	—
*E. procerum*	ME Noordeloos 2004070	Australia: Tasmania	—	GQ289183	GQ289254	GQ289323
*E. prunuloides*	MEN 200340	Slovakia	KC710073	GQ289184	GQ289255	GQ289324
*E. pseudofavrei*	JVG 1060930-7	—	JX454886	—	—	—
*E. pygmaeopapillatum*	ME Noordeloos 200364	Slovakia	—	GQ289185	GQ289256	GQ289325
*E. quadratum*	GDGM 28953	China: Jiangxi	—	KJ648471	KP226183	—
*E. radicipes*	KK 42/14, holotype	Finland	LN850585	—	LN850693	—
*E. readiae*	ME Noordeloos 2004050	Tasmania: Australia	—	GQ289186	GQ289257	GQ289326
*E. reductum*	SAAS 1016	China: Sichuan	**KU312117**	**KU534236**	**KU534482**	**KU534435**
*E. reductum*	SAAS 1091, holotype	China: Yunnan	**KU312123**	**KU534232**	**KU534480**	**KU534419**
*E. reductum*	SAAS 1608	China: Yunnan	**KU312124**	**KU534233**	**KU534481**	**KU534431**
*E. rhodocylix*	K (M): 147598	United Kingdom: Wales	KJ001415	KJ001450	—	—
*E. rhodopolium*	KK 1664/12	Sweden	LN850497	LN850705	LN850705	—
*E. rivulare*	KK 703/12, holotype	Finland	LN850544	LN850701	LN850707	—
*E. rubropilosum*	SAAS 406	China: Sichuan	MH020761	KU534218	KU534488	KU534439
*E. rusticoides*	LIP JVG 1020416U, epitypus	Spain: Tarragona	KJ001434	KJ001478	—	—
*E. sarcitum*	A. Hausknecht 1994-04-20	Austria	—	GQ289188	GQ289259	GQ289328
*E. saussetiense*	G. Eyssartier 08-067, holotype	France	LN850594	—	—	—
*E. sericatum*	KK 299/08	Finland	LN850442	—	LN850630	LN850702
*E. sericellum*	ME Noordeloos 200315	Belgium	—	GQ289190	GQ289261	GQ289330
*E. sericeonitidum*	TB 7144	—	EF421108	AF261315	EF421016	EF421108
*E. serpens*	KK 410/09, holotype	Finland	LN850526	—	LN850694	—
*E. serrulatum*	ME Noordeloos 2004062	Australia: Tasmania	—	GQ289192	GQ289263	GQ289332
*E. setastipes*	L.R. Hesler 13853, holotype	U.S.A.	LN850619	—	—	—
*E. sinuatum*	J. Wisman 2003-09-19	Netherlands	KC710109	GQ289193	GQ289264	GQ289333
*E. sordidulum*	Co-David 2003	Belgium	KC710062	GQ289194	GQ289265	GQ289334
*Entoloma* sp.	K389	Japan: Oita	AB691993	AB692008	AB692018	—
*Entoloma* sp.1	083001	China: Yunnan	**KU312119**	**KU534230**	**KU534479**	**KU534437**
*Entoloma* sp.1	SAAS 1154	China: Sichuan	**KU312121**	**KU534235**	**KU534478**	**KU534434**
*Entoloma* sp.2	SAAS 369	China: Jilin	**KU312104**	**KU534216**	**KU534483**	**KU534416**
*Entoloma* sp.3	SAAS 203	China: Jilin	KJ658966	KJ658971	KU534473	KU534415
*Entoloma* sp.4	SAAS 712	China: Shaanxi	KJ658970	KJ658973	KU534472	KU534417
*Entoloma* sp.5	SAAS 315	China: Sichuan	—	—	—	—
*E. subcaesiocinctum*	SAAS 133	China: Jilin	KY711236	KY972697	—	—
*E. sublaevisporum*	LIP JVG 1070823T, holotype	Spain	KC898436	KC898518	—	KC898478
*E. subtenuicystidiatum*	GDGM 28459	China: Jiangxi	JQ320109	JQ320116	—	JQ993071
*E. tectonicola*	P. Manimohan (741, holotype)	India	—	GQ289196	—	GQ289336
*E. tenellum*	Dhancke 2820	—	JX454933	—	—	—
*E. tenuissimum*	GDGM 28813	China: Jilin	JX975295	JQ993097	JQ993086	JQ993059
*E. terreum*	Esteve-Raventós et al. 16.X.2003, holotype	Spain	LN850547	—	—	—
*E. tjallingiorum*	S. Ryman (6124) (UPS: BOT: F-016378, holotype)	Sweden	KC898412	KC898509	—	KC898474
*E. trachyosporum*	H. den Bakker 1901	Canada	KC710121	GQ289199	—	GQ289339
*E. transmutans*	ME Noordeloos 2004155	Australia: Tasmania	—	GQ289200	GQ289268	GQ289340
*E. turbidum*	MEN 200351	Slovakia	KC710060	GQ289201	GQ289269	GQ289269
*E. undatum*	ME Noordeloos 200327	Belgium	—	GQ289202	GQ289270	GQ289342
*E. undatum*	JVG 1051115-19	Spain: Girona	KJ001410	KJ001455	—	—
*E. undulatosporum*	SFC 11021902	Spain: Barcelona	KJ001412	KJ001454	—	—
*E. valdeumbonatum*	M. Meusers E4565, holotype	Germany	—	GQ289203	GQ289271	GQ289343
*E. venustum*	L, Wö E17/10, holotype	Germany	KC898355	KC898523	—	KC898490
*E. vindobonense*	Wu 20810, holotype	—	JX454802	—	—	—
*E. violaceovillosum*	P. Manomohan 645, holotype	India: Kerala	—	GQ289205	GQ289273	GQ289345
*E. violaceozonatum*	V. Liiv (L 275, holotype)	Estonia	KC898448	KC898502	—	KC898467
*Inocephalus hypipamee*	DLL 10071	Australia	—	JQ624609	JQ624616	JQ624604
*Inocephalus plicatus*	DLL 10216	Australia	—	JQ624615	JQ624623	JQ624606
*Inocephalus* sp. 1	MCA 2479	—	—	GU384622	GU384640	GU384593
*Inocephalus* sp. 2	GD-b	Argentina	DQ490636	DQ457683	DQ472728	—
*Inocephalus* sp. 3	MCA 1867	—	—	GU384621	GU384638	GU384591
*Leptonia* sp.	MCA 1486	—	—	GU384623	GU384635	GU384589
*Lyophyllum leucophaeatum*	—	—	AF357032	AF223202	DQ367434	AF357101
*Nolanea sericea*	VHAs 03/02	—	—	DQ367423	DQ367435	EF421099
*N. strictior*	DUKE-JM96/10	—	EF421109	—	EF421017	EF421100
*Pouzarella albostrigosa*	DL Largent 9641	Australia: Queensland	—	HQ876535	HQ876513	HQ876557
*P. farinosa*	DL Largent 9934, holotype)	Australia: Queensland	—	HQ876516	HQ876495	HQ876538
*P. lasia*	DL Largent 9662	Australia: Queensland	—	HQ876529	HQ876507	HQ876551
*P. pilocystidiata*	DL Largent 9932, holotype	Australia: Queensland	—	HQ876521	HQ876500	HQ876543

Sequences in bold are newly generated in this study.

**Figure 1.** Basidiomes of *Claudopus* species. **a** Basidiomes of *E. conchatum* on soil (SAAS 1712) **b** Basidiomes of *E. conchatum* on stem of live *Pinus* (SAAS 1014) **c** Pileus of *E. flabellatum* (SAAS 1501) **d** Lamellae of *E. flabellatum* (SAAS 1080). **e** Basidiomes of *C. gregarium* on bark-wood of live *Castanopsis* (SAAS 1220) **f** Red droplets on the lamellar edges of *E. gregarium* (SAAS 1493) **g** Basidiomes of *E. pleurotoides* on decaying bark-wood of *Castanopsis* (SAAS 1215) **h** Basidiomes of *E. pleurotoides* on bark-wood of live *Castanopsis* (SAAS 1252) **i** Basidiomes of *E. reductum* on decaying stump of *Castanopsis* (SAAS 1091) **j** Mature basidiomes of *E. reductum* on rock (SAAS 2068) **k** Young basidiomes of *E. reductum* on soil (SAAS 1016) **l** Lamellae of E. byssisedum var. microsporum (SAAS 1828) **m** Basidiomes of E. byssisedum var. microsporum on decaying stump of *Betula* (SAAS 1160).

**Figure 2**. Microscopic structures of *Entoloma conchatum* (holotype): **a** Basidiospores **b** Pileipellis.

**Figure 3.** Microscopic structures of *Entoloma flabellatum* (holotype). **a** Basidiospores **b** Pileipellis.

**Figure 4.** Microscopic structures of *Entoloma gregarium* (holotype). **a** Basidiospores **b** Pileipellis.

**Figure 5.** Microscopic structures of *Entoloma pleurotoides* (holotype). **a** Basidiospores **b** Pileipellis.

**Figure 6.** Microscopic structures of *Entoloma reductum* (holotype). **a** Basidiospores **b** Pileipellis.

**Figure 7.** Microscopic structures of Entoloma byssisedum var. microsporum (SAAS 1279). **a** Basidiospores **b** Pileipellis.

